# The tRNA methyltransferase TrmB is critical for *Acinetobacter baumannii* stress responses and pulmonary infection

**DOI:** 10.1128/mbio.01416-23

**Published:** 2023-08-17

**Authors:** Jenna C. McGuffey, Clay D. Jackson-Litteken, Gisela Di Venanzio, Aubree A. Zimmer, Jessica M. Lewis, Jesus S. Distel, Kyusik Q. Kim, Hani S. Zaher, Juan Alfonzo, Nichollas E. Scott, Mario F. Feldman

**Affiliations:** 1 Department of Molecular Microbiology, Washington University School of Medicine in St. Louis, St. Louis, Missouri, USA; 2 Department of Microbiology and The Center for RNA Biology, The Ohio State University, Columbus, Ohio, USA; 3 Department of Microbiology and Immunology, The Peter Doherty Institute for Infection and Immunity, University of Melbourne, Melbourne, Victoria, Australia; 4 Department of Biology, Washington University in St. Louis, St. Louis, Missouri, USA; University of Georgia, Athens, Georgia, USA

**Keywords:** tRNA modification, *Acinetobacter*, iron acquisition, pneumonia, oxidative stress, macrophages

## Abstract

Successful pathogens must be able to adapt to a multitude of stressors imposed by their host. *Acinetobacter baumannii* has emerged as a major global health threat due to its exceptional ability to adapt to hostile environments and skyrocketing rates of multidrug resistance. Recent studies have begun to explore the importance of tRNA methylation in the regulation of bacterial stress responses, including adaptation to antibiotic and oxidative stresses. However, tRNA methyltransferases (trms) have not been investigated in *A. baumannii*. Bioinformatic analyses revealed nine putative, SAM-dependent trms conserved across clinical *A. baumannii* isolates and laboratory strains. We generated eight *trm* mutants in a modern, colistin-resistant clinical isolate, ARC6851, and analyzed the mutants’ stress responses. One mutant, Δ*trmB*, was vastly more sensitive to oxidative stress and displayed a growth defect at low pH. Accordingly, Δ*trmB* was unable to replicate in J774A.1 macrophages and had decreased virulence in an acute pneumonia murine model. Subsequently, we showed that *A. baumannii* TrmB makes the m^7^G tRNA modification. A proteomic analysis revealed that ARC6851 significantly upregulates a siderophore biosynthesis and uptake cluster, acinetobactin, under oxidative stress. In contrast, the upregulation of the acinetobactin proteins in Δ*trmB* was only modest, which impacted its ability to withstand iron deprivation under oxidative stress. qRT-PCR data showed that TrmB-dependent regulation of acinetobactin is post-transcriptional. Our results indicate that TrmB-mediated stress responses are important for *A. baumannii* pathogenesis, highlighting the therapeutic potential of targeting trms to combat the rise of multidrug-resistant *A. baumannii*.

**IMPORTANCE:**

As deficiencies in tRNA modifications have been linked to human diseases such as cancer and diabetes, much research has focused on the modifications’ impacts on translational regulation in eukaryotes. However, the significance of tRNA modifications in bacterial physiology remains largely unexplored. In this paper, we demonstrate that the m^7^G tRNA methyltransferase TrmB is crucial for a top-priority pathogen, *Acinetobacter baumannii*, to respond to stressors encountered during infection, including oxidative stress, low pH, and iron deprivation. We show that loss of TrmB dramatically attenuates a murine pulmonary infection. Given the current efforts to use another tRNA methyltransferase, TrmD, as an antimicrobial therapeutic target, we propose that TrmB, and other tRNA methyltransferases, may also be viable options for drug development to combat multidrug-resistant *A. baumannii*.

## INTRODUCTION

*Acinetobacter baumannii* is an opportunistic, Gram-negative pathogen that causes a wide range of nosocomial infections, including catheter-associated urinary tract infections (CAUTIs), endocarditis, meningitis, wound and burn infections, and, most commonly, ventilator-associated pneumonia and bacteremia (1). Notably, *A. baumannii* is acquiring multidrug resistance (MDR) phenotypes at unprecedented rates (2). In response, the World Health Organization has categorized carbapenem-resistant *A. baumannii* as a critical priority for research and development of new antibiotics (3). Unfortunately, this crisis has worsened during the COVID-19 pandemic, with rates of carbapenem-resistant *A. baumannii* rising 78% from 2019 to 2020 (4).

Over the past decade, research has failed to reveal one specific toxin or molecular mechanism that can explain the virulence potential of a particular *A. baumannii* strain (5). Therefore, our current understanding of *A. baumannii* pathogenesis relies on the pathogen’s ability to survive in and adapt to a wide variety of adverse conditions encountered during infection and in a healthcare environment, such as desiccation, disinfectants, oxidative stress, and iron deprivation (6). Unfortunately, the regulation of *A. baumannii* stress responses is still poorly understood.

To adapt to stressful conditions, several bacteria have recently been reported to use tRNA methylation to post-transcriptionally regulate the translation of transcripts that are enriched with specific mRNA codons (7 – 9). However, our understanding of the role of tRNA modifications in bacterial physiology and stress responses has barely scratched the surface. In fact, the full set of genes encoding tRNA modification enzymes has only been identified in *Escherichia coli* and *Mycoplasma capricolum* (10, 11). Furthermore, these identified genes are not conserved across all bacterial species, and it has been estimated that only 70%–80% of tRNA modifications can be predicted using homology to known tRNA modification enzymes (12). Additional diversity in the variety of tRNA modifications, or the tRNA “modificome,” stems from the identification of new modifications in other species that are not present in *E. coli,* such as the acetylated 3-(3-amino-3-carboxypropyl)uridine modification found in *Vibrio cholerae* (13). Furthermore, homologous tRNA modification enzymes may have different RNA targets in different species, and different species may use non-orthologous enzymes to either make the same modification or perform a similar function through distinct mechanisms (14 – 18). Importantly, these modifications can also be dependent on cell physiology and environmental conditions, as organisms have evolved unique solutions and require particular modifications according to the stressors that they encounter in their niches (7).

Given the diversity of tRNA modifications across bacterial species and the dearth of tRNA modificome research in many prokaryotes, it is imperative to identify the tRNA modifications present in human pathogens and elucidate their role in the pathogens’ abilities to respond to environmental stressors. Many tRNA modification pathways include a methylation step, with tRNAs being the most heavily methylated molecules in all domains of life (19, 20). In fact, tRNA methylation has been shown to impact bacterial stress responses, including antibiotic stress responses (8). Masuda et al. have shown that increased m^1^G37 levels in *E. coli* and *Salmonella* enhance the translation of mRNAs for membrane-associated proteins involved in barrier and efflux activity, preventing the intracellular accumulation of antimicrobial drugs and promoting the development of resistance (8). This modification is performed by the essential, conserved tRNA methyltransferase (trm) TrmD, an enzyme that has been widely targeted by pharmaceutical companies for the development of antimicrobials (21 – 23). Post-transcriptional regulation via trms also contributes to other bacterial stress responses; in *Pseudomonas aeruginosa*, the m^7^G46 modification catalyzed by TrmB influences the oxidative stress response via regulation of the *katA* and *katB* catalases (9). Exposure to H_2_O_2_ leads to increased levels of m^7^G methylation and increased translation efficiency of the Phe- and Asp- enriched mRNAs, including *katA* and *katB* transcripts. Accordingly, inactivation of *trmB* sensitizes *P. aeruginosa* to oxidative stress. Another modification, 2′-O-methylation of tRNA^Tyr^ catalyzed by *E. coli* TrmH may play a role in suppressing the host immune system response to bacterial tRNAs, potentially due to the modification reducing TLR7-mediated interferon release (24, 25).

While the field of bacterial tRNA modifications has begun to grow in recent years, the role of trms in *A. baumannii* remains elusive, and the *A. baumannii* tRNA modificome has not been identified. Several papers have suggested that methyltransferases could play a role in the rise of tigecycline resistance in *A. baumannii* clinical isolates; in the MDR *A. baumannii* isolate MDR-ZJ06, *trmB* was upregulated twofold in response to tigecycline stress (26). Additionally, two studies observed that disruption of another putative methyltransferase, A1S_2858, increased resistance to tigecycline in multiple strains (27, 28). However, these putative methyltransferases have not been functionally characterized in *A. baumannii*, and their roles in response to other stresses this bacterium may encounter during its pathogenesis and persistence have not been explored. Our lab has shown that *A. baumannii* utilizes unique pathways to respond to antibiotic and oxidative stresses; therefore, it is likely that *A. baumannii* may utilize its tRNA modificome in novel ways (29).

To explore the role of trms in *A. baumannii* stress responses and pathogenesis, we have identified nine, putative, SAM-dependent trms in the core *A. baumannii* genome and constructed deletion mutants. We report the mutants’ responses to antibiotic and oxidative stresses and demonstrate that TrmB is essential for complete oxidative and acid stress responses, macrophage infection, and virulence in a pneumonia murine model. We also provide whole-cell proteomic data under oxidative stress to gain insight into the role TrmB plays in *A. baumannii* physiology.

## RESULTS

### Nine putative SAM-dependent tRNA methyltransferases are conserved in *A. baumannii*

The presence, variety, and roles of trms in *A. baumannii* have not been explored. Basic Local Alignment Search Tool (BLAST) analyses using the sequences of trms from other bacteria revealed nine putative, SAM-dependent trms conserved across modern clinical *A. baumannii* isolates and laboratory strains (Table 1). Seven of these trms were annotated with functions previously identified in the literature. However, two putative trms did not have annotated targets, so we designated them trmZ1 and trmZ2; Phyre^2^ predicts that trmZ1 potentially catalyzes the formation of 5-methyluridine and trmZ2 may act as a 7-methylguanosine methyltransferase or a 2′-O-methyltransferase (30). Many of these trms have not been fully characterized in bacteria, and their activities and targets may be species-specific (12). Therefore, we generated unmarked trm deletion mutants in the modern clinical isolate ARC6851, a particularly virulent respiratory pathogen that is resistant to the last-resort antibiotic colistin (Table 1). Ultimately, we were able to generate unmarked mutants for all putative SAM-dependent *A. baumannii* trms except *trmD*, which catalyzes the essential m^1^G37 modification conserved across all domains of life (31).

**TABLE 1:** ARC6851 has nine putative, SAM-dependent tRNA methyltransferases^*a*^

Gene	Predicted modification	ARC6851 locus tag
*trmA*	m^5^U (54)	OB946_10490
*trmB*	m^7^G (46)	OB946_12575
*mnmC*	mnm^5^U (34)	OB946_02580
*trmD*	m^1^G (37)	OB946_01600
*trmJ*	2′OmC/U	OB946_10840
*trmL*	2′OmC (34)	OB946_03595
*trmO*	m^6^t^6^A (37)	OB946_06360
*trmZ1*	m^5^U	OB946_02910
*trmZ2*	m^7^G	OB946_16835

^*a*^ The prediction modifications are denoted as follows: m^5^U (54) (5-methyluridine at position 54), m^7^G (46) (7-methylguanosine at position 46), mnm^5^U (34) (5-methylaminomethyl uridine at position 34), m^1^G (37) (1-methylguanosine at position 37), 2′OmC/U (2′-O-methylcytidine or 2′-O-methyluridine), 2′OmC (34) (2′-O-methylcytidine at position 34), m^6^t^6^A (37) (N6-methyl-threonylcarbamoyladenosine at position 37), m^5^U (5-methyluridine), and m^7^G (7-methylguanosine).

### Loss of single trms does not significantly impact ARC6851 antibiotic susceptibility

**Fig 1:**
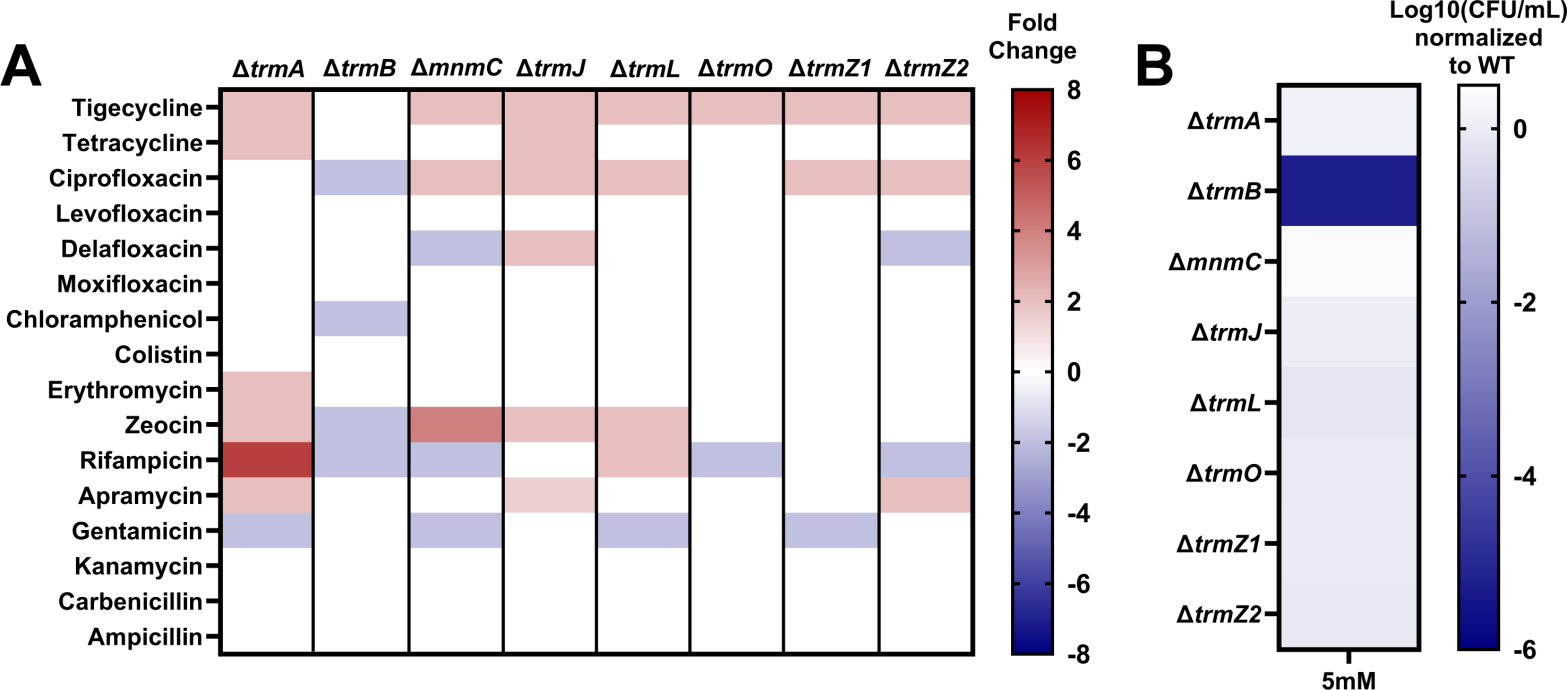
Loss of *trmB* does not significantly impact antibiotic susceptibility, but renders ARC6851 more susceptible to oxidative stress (**A**) Antibiotic susceptibility of *trm* mutants. Eight ARC6851 *trm* deletion mutants were screened for changes in MICs to a variety of antibiotics using a 2-fold broth dilution method. Mid-exponential cultures were normalized at OD_600_ 0.01 and grown for 16 h with shaking at 37°C. MIC was determined as <10% growth compared to a non-treated culture. Greater than 2-fold changes were considered significant. (**B**) Oxidative stress resistance of *trm* mutants. Strains were grown to mid-exponential phase before being treated with 0 mM or 5 mM H_2_O_2_ for 2 h. Survival was measured by serial dilution and quantification of the recoverable CFU/mL. Fold changes were calculated against the wild-type strain.

As trms mediate antibiotic resistance in several bacteria, we determined whether inactivation of the putative trms affected ARC6851 antibiotic susceptibility. In our screen, we included tigecycline and its ancestor tetracycline, as tigecycline was the only antibiotic associated with trm regulation in *A. baumannii* literature (26 – 28). In addition, we tested several quinolones that target DNA topoisomerases; chloramphenicol, erythromycin, and aminoglycosides that target protein synthesis; rifampicin which targets RNA polymerase; zeocin which cleaves DNA; beta-lactams that target cell-wall synthesis; and colistin which targets the cell membrane. Ultimately, we did not observe many significant alterations (defined here as >2-fold) in the minimum inhibitory concentrations (MICs) of this diverse array of antibiotics (Fig. 1A). Interestingly, Δ*trmA* had a MIC 4-fold higher for rifampicin. This phenotype was not seen in the other mutants, further suggesting that trms can be involved in modulating the responses to different stressors. While Δ*trmB* showed 2-fold lower MICs for several antibiotics, we did not observe any change in MIC to tigecycline or tetracycline, suggesting that *A. baumannii* TrmB may not play a role in tigecycline resistance. However, we did observe a 2-fold decrease in tigecycline susceptibility with the *trmZ1* mutant, the putative methyltransferase whose absence was reported to increase tigecycline resistance in *A. baumannii* strains (27, 28). Overall, these *trms* do not appear to play a major role in determining antibiotic susceptibility in *A. baumannii*.

### TrmB is essential for resistance to oxidative and acid stresses

Next, we assessed the roles of the trms in resistance to another environmental and host stressor, oxidative stress. A previous report in *P. aeruginosa* suggested that PaTrmB plays a role in its oxidative stress response (9). To investigate if trms mediate the *A. baumannii* response to oxidative stress, cells were treated with 5 mM H_2_O_2_. Strikingly, the Δ*trmB* mutant was almost entirely killed, while the other mutants behaved similarly to wild-type bacteria at this concentration (Fig. 1B). As opposed to *P. aeruginosa*, we did not observe the same phenotype in the Δ*trmJ* mutant, suggesting *A. baumannii* oxidative stress responses may differ (32). Given the Δ*trmB* mutant’s inability to survive oxidative stress, and the lack of data on the role of bacterial TrmB *in vivo* and in other stressful conditions, we decided to further investigate the role of TrmB in *A. baumannii* pathogenesis.

**Fig 2:**
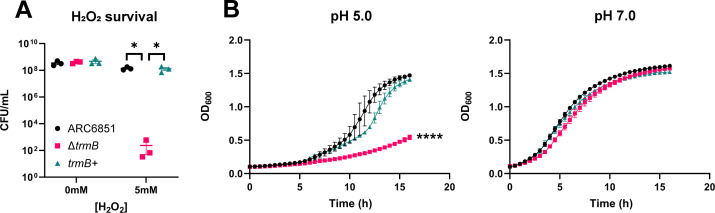
TrmB is essential for resistance to oxidative and acid stresses. (**A**) H_2_O_2_ killing of ARC6851 strains. Wild-type, Δ*trmB*, and *trmB*+ strains were grown to mid-exponential phase before being treated with 0 mM or 5 mM H_2_O_2_ for 2 h. Survival was measured by serial dilution and quantification of recoverable CFU/mL. Points represent technical replicates from at least three biological replicates, with a horizontal line representing the mean, and errors bars representing the standard error of the mean (SEM). *P < 0.05; one-way ANOVA, Tukey’s test for multiple comparisons. (**B and C**) Representative growth curves of wild type, Δ*trmB*, and *trmB*+ in non-buffered LB at pH 5.0 and pH 7.0. Mid-exponential cultures were normalized to OD_600_ = 0.01 and grown for 16 h with shaking at 37°C. ****P < 0.0001, unpaired *t* tests at 16 h for Δ*trmB* compared to wild type and *trmB*+.

We next complemented the Δ*trmB* mutant by chromosomally inserting a copy of the wild-type allele to validate our screening results, *trmB*+. Indeed, we found that the increased susceptibility to H_2_O_2_ was due to the loss of TrmB (Fig. 2A). Subsequently, we tested another major stressor encountered during pathogenesis, acid stress. As a byproduct of its metabolism, *A. baumannii* secretes large amounts of ammonia, resulting in the alkalization of its environment, and our lab recently discovered that *A. baumannii* also alkalizes its vacuole within macrophages during intracellular replication (33). At pH 7.0, we observed that the mutant is significantly shorter in length than the wild-type cells; however, we did not observe a growth defect (Fig S1; Fig. 2B). Therefore, we subjected the mutant to grow in a non-buffered, acidic, rich media. The mutant presented a growth defect that was successfully complemented (Fig. 2B; Fig S2). This defect suggests that the Δ*trmB* mutant may be at a disadvantage *in vivo* because replicative strains must be able to grow quickly at an acidic pH to produce ammonia that can neutralize the acidic vacuole, ultimately avoiding degradation.

### TrmB plays a significant role in bacterial replication within macrophages

**Fig 3:**
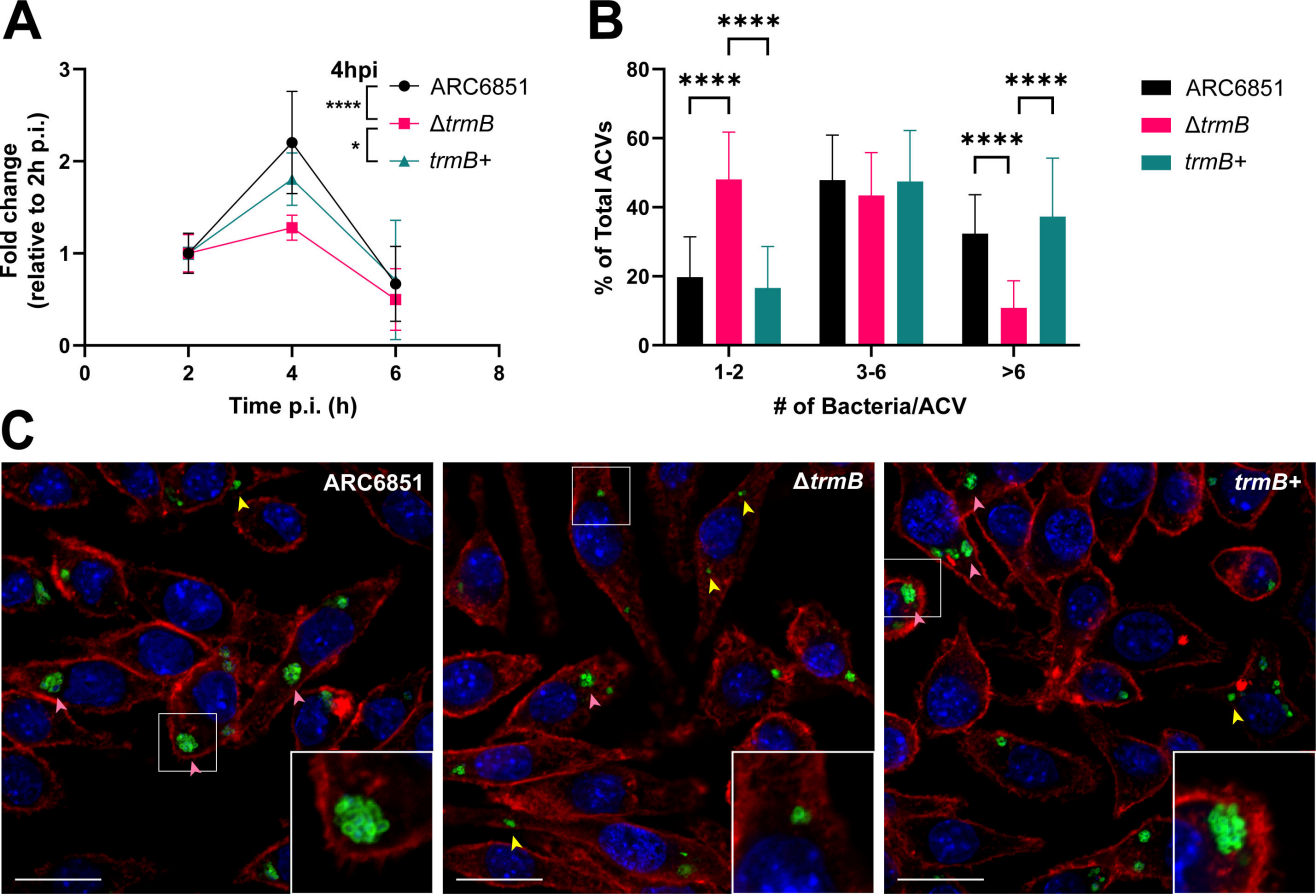
TrmB plays a significant role in replication within macrophages (**A**) J774A.1 macrophages were infected with mid-exponential phase ARC6851 wild-type, Δ*trmB*, and *trmB*+ strains. Intracellular CFU were determined at 2, 4, and 6 h post-infection. *P < 0.05, ****P < 0.0001, mixed effects model, Tukey’s test for multiple comparisons. (**B**) Infected macrophages were fixed at 4 h post-infection. Number of bacteria per ACV were determined with at least 14 representative confocal microscopy images and two biological replicates per strain. (**C**) The samples were stained to detect cell nuclei (blue), *A. baumannii* (green), and actin (red). Bars 20 µM. Insets (13 µM) are a higher magnification of the area denoted in the white box of the corresponding image. Representative ACVs are indicated with pink (>six bacteria/ACV) and yellow (one to two bacteria/ACV) arrows.

As pathogens encounter both oxidative and acid stressors imposed by the host during infection, these data suggest that *trmB*-deficient ARC6851 may be at a disadvantage during pathogenesis. Previous work on bacterial TrmB has solely been performed *in vitro*, but the impact *in vivo* and in *A. baumannii* remains unknown. Macrophages contribute to host defense against *A. baumannii* infections, but our lab has recently shown that multiple new clinical *A. baumannii* isolates, unlike domesticated lab strains, can replicate inside spacious vacuoles within macrophages (34). Therefore, we first confirmed that ARC6851 can replicate inside J774A.1 macrophages and found that ARC6851 wild type doubles in J774A.1 macrophages by 4 h post-infection (Fig. 3A). Remarkably, the Δ*trmB* mutant had a replication defect in the macrophages, only persisting within the cells for 4 h. Using confocal microscopy, we also observed less bacteria per *A. baumannii*-containing vacuole (ACV) compared to the wild-type and complemented strains (Fig. 3B and C), while observing similar rates of infection and ACVs per cell (Fig S3). Altogether these data suggest that Δ*trmB* was able to be phagocytosed at similar rates as wild type, but was unable to replicate once inside its vacuole, potentially due to its inability to respond to the stresses imposed by the macrophages (i.e., oxidative and pH).

### ARC6851 Δ*trmB* is attenuated in an acute murine pneumonia model

**Fig 4:**
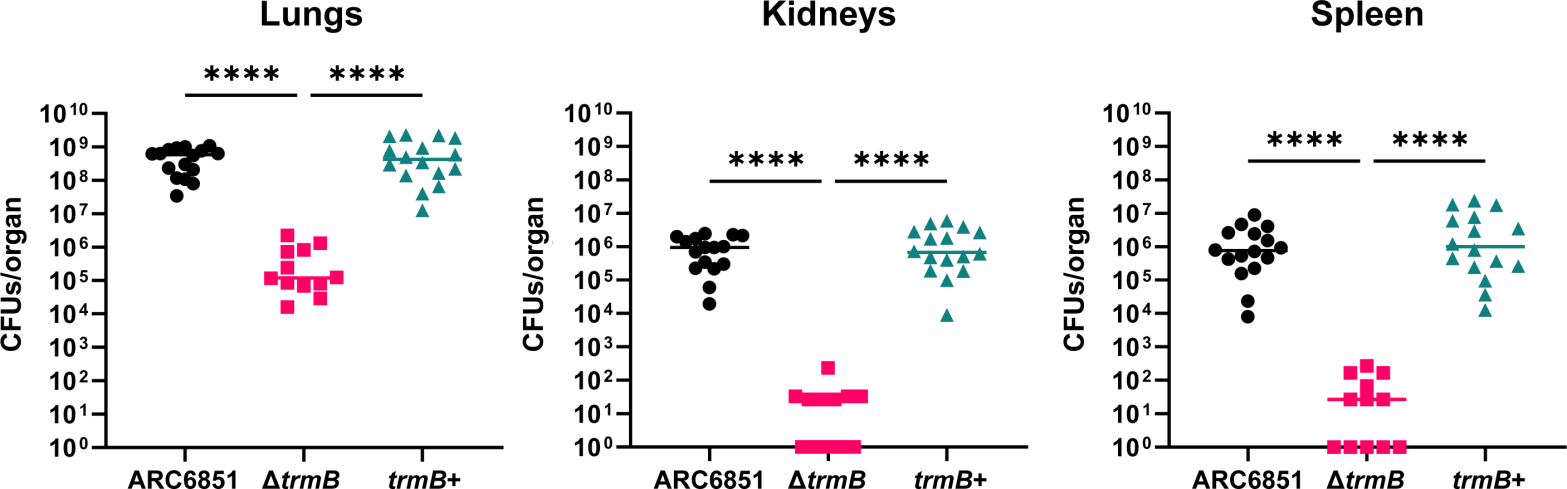
ARC6851 Δ*trmB* is attenuated in an acute murine pneumonia model. C57BL/6 mice were infected with ~5 × 10^7^ CFU of mid-exponential ARC6851 wild-type, Δ*trmB*, and *trmB*+ strains. At 24 h post-infection, the lungs, kidneys, and spleens were harvested, and the bacterial load present in each tissue was determined with serial dilutions. Each symbol represents an individual mouse, and the horizontal bar represents the median. Data collected from three independent experiments. ****P < 0.0001, Kruskal-Wallis test.

Given the role of macrophages during *A. baumannii* pneumonia infections and the mutant’s defect in J774A.1 macrophages, we next assessed whether *trmB* is required for virulence in an acute murine pneumonia model, as previously described (35). Briefly, mice were intranasally inoculated with wild-type, Δ*trmB*, and *trmB*+ strains, and at 24 h post-infection, the lungs, spleens, and kidneys were harvested and processed to determine bacterial burden. Ultimately, we observed that the Δ*trmB* mutant had a significant reduction (>10,000-fold) in recovered CFU as compared to the wild-type and complemented ARC6851 strains in all tested organs (Fig. 4).

While *A. baumannii* is most commonly associated with pneumonia, we recently found that one-fifth of isolates come from UTIs (36). Given that the lung and urinary tract environments significantly differ, we also decided to assess the role of TrmB in our recently developed CAUTI model (36). However, ARC6851 does not establish CAUTI in this model, so we generated Δ*trmB* and *trmB*+ strains in another modern MDR clinical isolate, Ab04. We found that Ab04 Δ*trmB* (ACX61_12415) is not attenuated in our murine CAUTI model, suggesting that the role of *trmB* may be specific to the stresses imposed by a pneumonia infection (Fig. S4).

### *A. baumannii* TrmB performs the m^7^G tRNA modification

**Fig 5:**
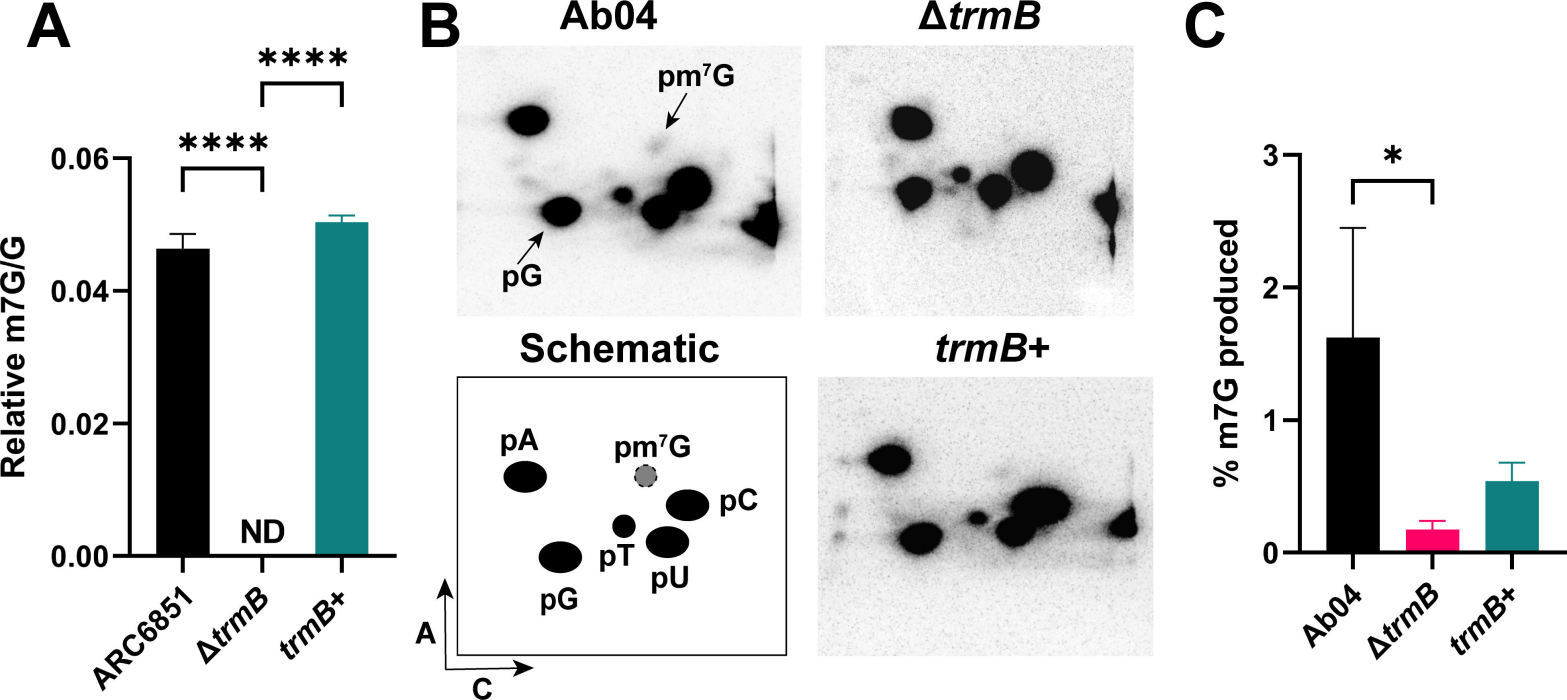
*A. baumannii* TrmB performs the m^7^G tRNA modification. (**A**) m^7^G levels in ARC6851 wild-type, Δ*trmB*, and *trmB*+ strains were determined with LC-MS. Δ*trmB* m^7^G levels were below detection limit (ND). ****P < 0.0001, one-way ANOVA, Tukey’s test for multiple comparisons. (**B**) m^7^G levels in Ab04 wild-type, Δ*trmB*, and *trmB*+ strains were determined with thin-layer chromatography and (**C**) analyzed with ImageQuant. *P < 0.05, one-way ANOVA, Tukey’s test for multiple comparisons.

As the sequence homology of TrmB is not sufficient to determine its function, we verified that TrmB makes the m ^7^ G modification in *A. baumannii* similarly to its homologs in other bacteria (9, 37, 38). For ARC6851, we used LC-MS to assess m^7^G levels in wild-type, Δ*trmB*, and *trmB*+ strains. As predicted, we found that Δ*trmB* lacks m^7^G, and the presence of m^7^G is restored in the complemented strain (Fig. 5A). For Ab04, we employed a different technique, thin-layer chromatography (TLC), to measure m^7^G levels. Similarly, we observed decreased levels of m^7^G in the mutant strain, demonstrating that TrmB does indeed make the m^7^G modification in multiple clinical *A. baumannii* isolates (Fig. 5B). The Ab04 phenotype was partially complemented; however, all strains have been whole genome sequenced to avoid additional mutations. Therefore, this partial complementation is likely due to the restored gene being located at a different site in the chromosome and lacking all of its native regulatory sequences.

### TrmB plays a significant regulatory role in ARC6851 under oxidative stress

**Fig 6:**
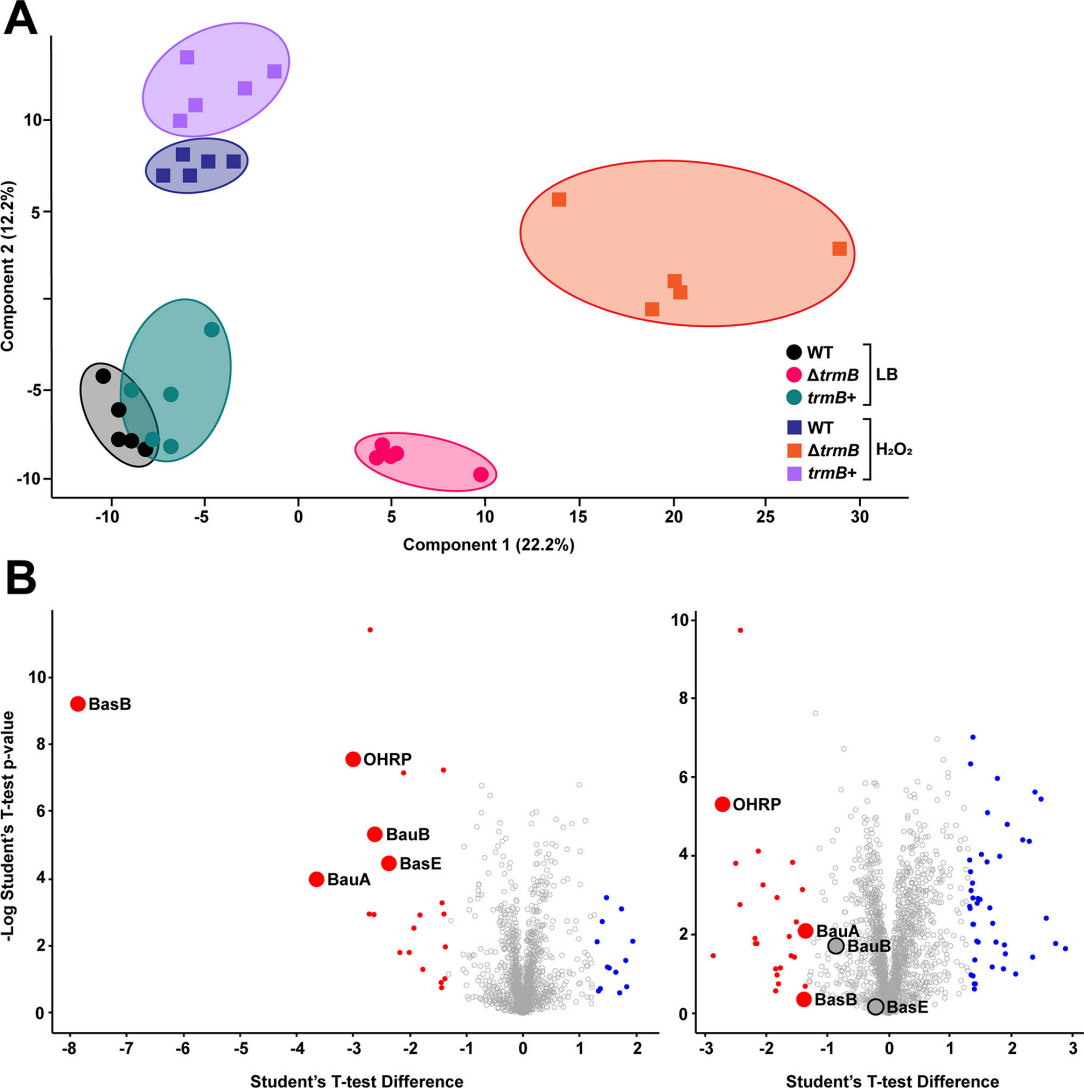
TrmB plays a significant regulatory role in ARC6851 under oxidative stress. (**A**) Principal component analysis of ARC6851 wild-type, Δ*trmB*, and *trmB*+ proteomes with and without H_2_O_2_ treatment. Mid-exponential cultures were treated with 0 mM or 5 mM H_2_O_2_ for 2 h before being pelleted, lysed, and acetone precipitated. Digested proteome samples were analyzed with reverse phase liquid chromatography-mass spectrometry. (**B**) (left) Volcano plot of Table 2 data showing differentially expressed proteins in ARC6851 wild type in H_2_O_2_ treatment versus non-treated. (right) Volcano plot of Table 3 data showing differentially expressed proteins in ARC6851 Δ*trmB* in H_2_O_2_ treatment versus non-treated. Red dots are more than 2.5-fold upregulated, and blue dots are more than 2.5-fold downregulated. Large dots represent hits used for further analysis.

tRNA modifications can affect the translation efficiency of mRNAs enriched for specific codons, which can be used to post-transcriptionally regulate the translation of stress-related factors (8, 9). To identify proteins affected by the presence or absence of m^7^G in ARC6851, we performed whole-cell proteomics of wild-type, Δ*trmB*, and *trmB*+ strains grown with and without H_2_O_2_ treatment. Notably, the principal component analysis showed distinct grouping of the wild-type and complemented strains with and without stress, while the mutant clustered separately in both conditions (Fig. 6). This analysis indicates that the Δ*trmB* mutant has global changes in its translation profiles in the presence or absence of H_2_O_2_ as compared to the wild-type and complemented strains.

**TABLE 2:** Differentially expressed proteins in ARC6851 wild type in H_2_O_2_ treatment vs non-treated

Accession	Fold change^*a*^	Annotated protein
UYC79050.1	**226.13**	Acinetobactin non-ribosomal peptide synthetase subunit BasB
UYC78208.1	**12.74**	TonB-dependent siderophore receptor BauA
UYC76661.1	**8.05**	Organic hydroperoxide resistance protein
UYC79121.1	**6.57**	TonB-dependent receptor
UYC75768.1	**6.47**	Alkyl hydroperoxide reductase subunit F
UYC78207.1	**6.21**	Siderophore-binding periplasmic lipoprotein BauB
UYC76254.1	**6.20**	TonB-dependent siderophore receptor
UYC78211.1	**5.14**	(2,3-Dihydroxybenzoyl)adenylate synthase BasE
UYC77454.1	**4.52**	Amino acid permease
UYC75593.1	**4.32**	Acetyltransferase
UYC78132.1	**4.03**	Type I-F CRISPR-associated helicase Cas3f
UYC76981.1	**3.80**	Ferrous iron transport protein A
UYC76313.1	**3.52**	Biliverdin-producing heme oxygenase
UYC78228.1	**3.40**	Xanthine dehydrogenase molybdopterin binding subunit
UYC76709.1	2.72	RidA family protein
UYC75664.1	2.71	PilZ domain-containing protein
UYC78065.1	**2.68**	Sulfate ABC transporter ATP-binding protein
UYC76832.1	**2.64**	Catalase/peroxidase HPI
UYC78212.1	**2.63**	Acinetobactin biosynthesis bifunctional isochorismatase/aryl carrier protein BasF
UYC78043.1	2.60	Protein TolR
UYC78131.1	**2.58**	Type I-F CRISPR-associated endonuclease Cas1f
UYC77811.1	**0.40**	RtcB family protein
UYC77797.1	0.40	Hypothetical protein OB946_02925
UYC78848.1	0.39	Muconolactone Delta-isomerase
UYC78838.1	**0.38**	PDR/VanB family oxidoreductase
UYC79075.1	**0.36**	Acyl-CoA dehydrogenase family protein
UYC77864.1	**0.35**	Asp-tRNA(Asn)/Glu-tRNA(Gln) amidotransferase subunit GatC
UYC78708.1	**0.35**	Hypothetical protein OB946_07885
UYC79007.1	0.32	MFS transporter
UYC76575.1	0.31	Cold-shock domain-containing protein
UYC78839.1	**0.30**	Flavin reductase family protein
UYC78097.1	**0.28**	MFS transporter
UYC78900.1	0.28	TetR/AcrR family transcriptional regulator
UYC76833.1	**0.26**	MBL fold metallo-hydrolase

^*a*^ Fold change cutoff: 2.5-fold, bolded if *P* value < 0.05, Student’s unpaired *t* test.

**TABLE 3:** Differentially expressed proteins in ARC6851 Δ*trmB* in H_2_O_2_ treatment vs non-treated

Accession	Fold change^*a*^	Annotated protein
UYC78524.1	**7.32**	TetR/AcrR family transcriptional regulator
UYC76661.1	**6.74**	Organic hydroperoxide resistance protein
UYC78132.1	**5.65**	Type I-F CRISPR-associated helicase Cas3f
UYC76254.1	**5.38**	TonB-dependent siderophore receptor
UYC75768.1	**5.37**	Alkyl hydroperoxide reductase subunit F
UYC78695.1	**4.53**	GTP 3,8-cyclase MoaA
UYC76045.1	**4.51**	Cold-shock protein
UYC78239.1	**4.46**	Hypothetical protein OB946_05355
UYC78349.1	**4.38**	Hypothetical protein OB946_05940
UYC77774.1	**4.15**	Acyl-CoA desaturase
UYC76578.1	3.60	NADH-quinone oxidoreductase subunit M
UYC76099.1	**3.59**	Ribonuclease HII
UYC76290.1	**3.55**	Poly-beta-1,6-N-acetyl-D-glucosamine biosynthesis protein PgaD
UYC78650.1	3.54	ATP-binding cassette domain-containing protein
UYC78900.1	3.48	TetR/AcrR family transcriptional regulator
UYC78238.1	3.41	Hypothetical protein OB946_05350
UYC76938.1	**3.08**	TRAP transporter large permease subunit
UYC77398.1	**3.02**	DUF1737 domain-containing protein
UYC75593.1	**2.96**	Acetyltransferase
UYC78982.1	**2.91**	Uracil-DNA glycosylase family protein
UYC78065.1	**2.85**	Sulfate ABC transporter ATP-binding protein
UYC78208.1	**2.73**	TonB-dependent siderophore receptor BauA
UYC77179.1	**2.66**	GntR family transcriptional regulator
UYC76950.1	2.59	Preprotein translocase subunit SecE
UYC79112.1	2.57	Hypothetical protein OB946_12705
UYC79050.1	2.55	Acinetobactin non-ribosomal peptide synthetase subunit BasB
UYC77830.1	**0.40**	YegP family protein
UYC75549.1	**0.40**	Thiamine pyrophosphate-dependent dehydrogenase E1 component subunit alpha
UYC76443.1	**0.40**	Gamma-glutamyltransferase
UYC77151.1	**0.40**	Enoyl-CoA hydratase
UYC78302.1	**0.40**	Alpha/beta hydrolase
UYC75929.1	**0.39**	1,2-phenylacetyl-CoA epoxidase subunit A
UYC78495.1	0.39	SRPBCC family protein
UYC75928.1	**0.39**	1,2-phenylacetyl-CoA epoxidase subunit B
UYC78130.1	**0.39**	DUF962 domain-containing protein
UYC77150.1	**0.39**	Enoyl-CoA hydratase/isomerase family protein
UYC78788.1	**0.38**	Hypothetical protein OB946_08315
UYC75753.1	0.38	Tautomerase family protein
UYC75847.1	**0.38**	Class I SAM-dependent methyltransferase
UYC76799.1	0.38	RNA-binding protein
UYC75632.1	0.38	Hypothetical protein OB946_10145
UYC76150.1	**0.38**	Cache domain-containing protein
UYC78477.1	0.38	DUF2171 domain-containing protein
UYC78379.1	**0.37**	Hypothetical protein OB946_06090
UYC78033.1	**0.37**	Rhombotarget A
UYC77971.1	**0.36**	Diacylglycerol kinase family protein
UYC77152.1	**0.36**	Acyl-CoA dehydrogenase family protein
UYC78876.1	**0.36**	Hydrolase
UYC78647.1	**0.35**	OmpW family protein
UYC77692.1	**0.33**	Ribonuclease I
UYC77169.1	**0.33**	SDR family NAD(*P*)-dependent oxidoreductase
UYC75548.1	**0.32**	Alpha-ketoacid dehydrogenase subunit beta
UYC78437.1	0.31	YeaC family protein
UYC77309.1	**0.31**	DNA-3-methyladenine glycosylase I
UYC75917.1	**0.30**	PaaI family thioesterase
UYC78152.1	**0.29**	Thiamine pyrophosphate-binding protein
UYC79075.1	**0.28**	Acyl-CoA dehydrogenase family protein
UYC77165.1	0.27	Ester cyclase
UYC78031.1	**0.27**	AI-2E family transporter
UYC76604.1	**0.27**	TetR/AcrR family transcriptional regulator
UYC78839.1	**0.26**	Flavin reductase family protein
UYC76941.1	0.24	YMGG-like glycine zipper-containing protein
UYC77154.1	**0.22**	3-hydroxyisobutyrate dehydrogenase
UYC77153.1	**0.20**	AMP-binding protein
UYC78778.1	**0.20**	Rieske (2Fe-2S) protein
UYC77155.1	**0.19**	CoA-acylating methylmalonate-semialdehyde dehydrogenase
UYC78838.1	**0.18**	PDR/VanB family oxidoreductase
UYC78767.1	**0.17**	Hypothetical protein OB946_08205
UYC78848.1	**0.15**	Muconolactone Delta-isomerase
UYC77084.1	**0.14**	RidA family protein

^*a*^ Fold change cutoff: 2.5-fold, bolded if *P* value < 0.05, Student’s unpaired *t* test.

**Fig 7:**
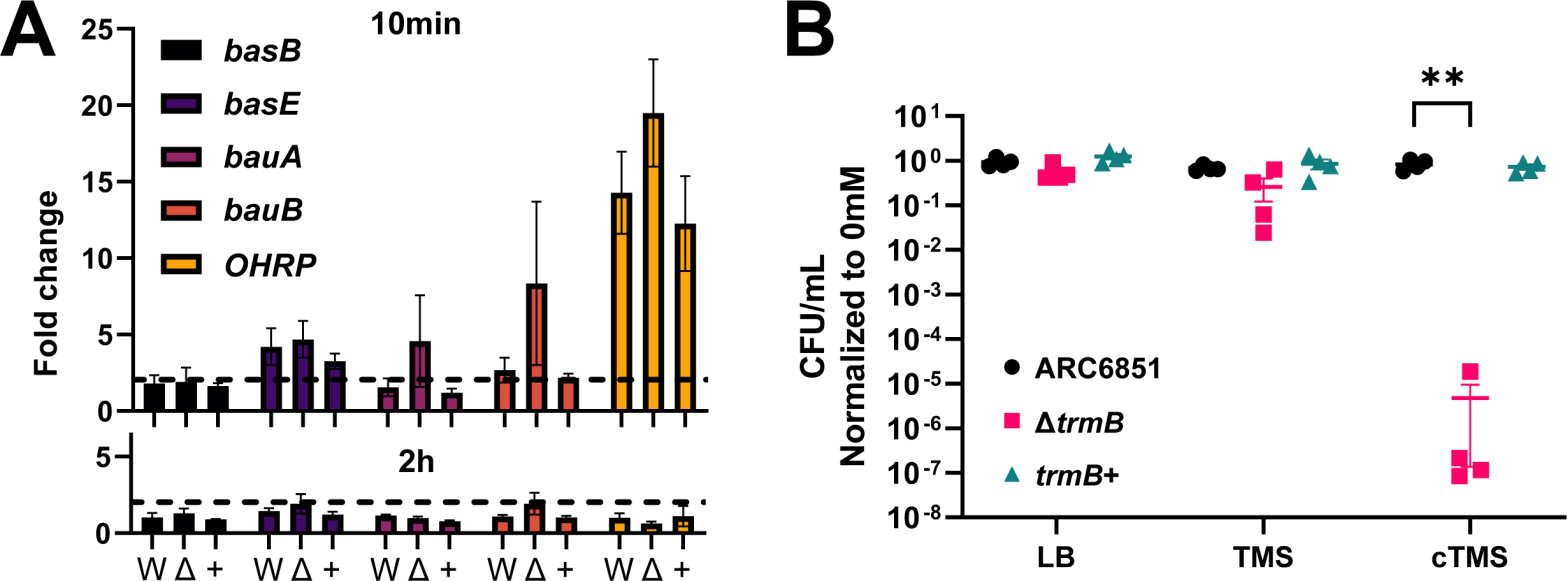
ARC6851 Δ*trmB* fails to post-transcriptionally upregulate acinetobactin and is more susceptible to iron deprivation under H_2_O_2_ stress. (**A**) Relative gene expression of *basB*, *basE*, *bauA*, *bauB*, and OB946_15780 (*OHRP*) genes in ARC6851 wild-type (W), Δ*trmB* (Δ), and *trmB*+ (+) strains grown in 2 mM H_2_O_2_ for 10 min (top) or 2 h (bottom) compared to 0 mM, as determined by qRT-PCR. Dotted line represents 2-fold change. (**B**) H_2_O_2_ killing of ARC6851 wild-type, Δ*trmB*, and *trmB*+ strains in grown in LB, Tris minimal succinate (TMS) media, or Chelex-100-treated TMS (cTMS) with treatment in 3 mM H_2_O_2_ for 2 h compared to 0 mM. Survival was measured by serial dilution and quantification of recoverable CFU/mL. Points represent technical replicates from at least four biological replicates, with a horizontal line representing the mean, and errors bars representing the standard error of the mean (SEM). **P < 0.01; one-way ANOVA, Tukey’s test for multiple comparisons.

Functional analysis of the proteomic data revealed that under H_2_O_2_-imposed stress ARC6851 upregulates many proteins involved in inorganic ion transport and metabolism, while the Δ*trmB* mutant upregulates more proteins involved in transcription and replication, potentially to replace damaged proteins (Fig S5). Under oxidative stress, we observed a putative catalase (UYC76832.1) was upregulated 2.6-fold in the wild type but only 1.8-fold in the mutant (Supplemental data set). While this minor difference may contribute to the Δ*trmB* mutant’s large increase in susceptibility to hydrogen peroxide, it is unlikely to be the sole contributor to this phenotype. Notably, we observed that in response to H_2_O_2_, wild-type ARC6581 upregulates several members of a siderophore cluster, acinetobactin, previously reported to be required for *Acinetobacter* virulence (Table 2) (39 – 41). Strikingly, BasB, a putative acinetobactin biosynthesis protein, was upregulated ~200-fold in wild-type ARC6851 under oxidative stress but was only upregulated 2.6-fold in the Δ*trmB* mutant (Table 2; Supplemental data set). The other members of the cluster that were upregulated in wild-type ARC6851 include BauA (12.7-fold in wild type vs 2.7-fold in the Δ*trmB* mutant), BauB (6.2-fold vs 1.8-fold), and BasE (5.1-fold vs 1.0-fold). Juttukonda et al. reported that several acinetobactin biosynthesis genes are transcriptionally upregulated in the model, “domesticated” *A. baumannii* strain Ab17978 in response to H_2_O_2_-imposed stress, so we aimed to determine whether the large differences we observed in wild-type compared to the Δ*trmB* mutant in the ARC6851 clinical isolate were due to transcriptional or post-transcriptional regulation (42). We performed qRT-PCR on wild-type, Δ*trmB*, and complemented strains exposed to 2 mM H_2_O_2_ for 10 min to see immediate changes and 2 h to match the proteomic conditions. Surprisingly, we observed that the acinetobactin cluster genes *basB*, *basE*, *bauA*, and *bauB* were not more transcriptionally upregulated in wild type compared to the mutant at either 10 min or 2 h of oxidative stress, indicating that the large differences observed in the proteomics data set were due to post-transcriptional regulation (Fig. 7A).

As a proof of principle, we also tested the transcriptional levels of the most upregulated protein in wild type that was similarly upregulated in the mutant, an annotated organic hydroperoxide resistance protein (OHRP, UYC76661.1). Its corresponding gene, *OB946_15780*, was highly upregulated at 10 min for both wild type and the mutant, which may indicate that this response to the imposed stress was highly induced at the transcriptional level, in contrast to the acinetobactin cluster (Fig. 7A). This finding supports the hypothesis that the difference in TrmB-dependent oxidative stress responses is due to post-transcriptional regulation, while responses that occur in both wild-type and the Δ*trmB* mutant may involve transcriptional regulation.

These data led us to hypothesize that the Δ*trmB* mutant would be more susceptible to iron deprivation during H_2_O_2_-imposed stress since the strain is unable to efficiently upregulate key members of the acinetobactin cluster. To test this, we performed H_2_O_2_ killing assays at a sublethal concentration in rich or Tris-minimal succinate (TMS) media. When the Δ*trmB* mutant was exposed to H_2_O_2_ in TMS media treated with a chelating resin (cTMS), it was almost entirely killed, indicating that loss of *trmB* disrupted its ability to respond to iron-limiting conditions when also exposed to low levels of oxidative stress (Fig. 7B). As bacterial pathogens must withstand oxidative stressors and possess efficient mechanisms of iron acquisition to survive within a host and establish infection, the Δ*trmB* mutant’s increased susceptibility to iron deprivation during oxidative stress may explain its dramatic fitness defect during pneumonia.

## DISCUSSION

tRNA modifications are important for translation, and therefore to the physiology of pathogens that are prominent global health threats; however, research has been largely focused on eukaryotes, as tRNA modifications have been linked to a multitude of human diseases (43). It is now increasingly clear that different bacterial species use tRNA modifications in distinct and diverse ways. This form of post-transcriptional regulation quickly modulates the translation efficiency of proteins, particularly those that may be used to respond to stressors encountered during infection (12, 44, 45). As the genetic code is inherently degenerate and codon bias is unique to each species, it is likely that the tRNA species targeted by the trms can vary between species, impacting the translation of modified tunable transcripts (MoTTs) that are enriched for the corresponding codons and/or are sensitive to alteration in levels of tRNA modifications. While TrmB is important for the *P. aeruginosa* oxidative stress response, it is critical that we investigate the role of *trmB* in *A. baumannii*, given the pathogen’s unique virulence factors and stress response pathways.

A previous study has reported that *A. baumannii* Ab17978 transcriptionally upregulates the acinetobactin siderophore cluster, potentially in response to iron-sulfur clusters being oxidized during H_2_O_2_ treatment (42). In ARC6851, this upregulation appears to be post-transcriptional, and the Δ*trmB* mutant cannot upregulate the members of the acinetobactin cluster involved in biosynthesis and uptake to the same degree as the wild-type strain. As acinetobactin has also been shown to be required for *A. baumannii* virulence, the mutant’s inability to post-transcriptionally upregulate acinetobactin to appropriate levels may be one of the mechanisms by which loss of *trmB* leads to decreased pathogenesis, especially as iron acquisition is key to a pathogen’s success within a host (39 – 41). Interestingly, a recent report described a role for queuosine tRNA modifications during multimetal starvation in *A. baumannii,* further supporting the importance of exploring the impact tRNA modifications have on *A. baumannii* pathobiology (46).

Although the mechanism of TrmB in *A. baumannii* remains unknown, it is tempting to speculate that the presence of m^7^G may impact the stability of other modifications on the same tRNA. For example, the m^7^G46 modification has been shown to be critical for tRNA stability in several organisms, whereby bases G46, C13, and G22 make a base triple pair to maintain the canonical L structure (47 – 52). When a *Thermus thermophilus* Δ*trmB* mutant was exposed to high temperatures, many nucleotide positions became hypomodified and the strain had a severe growth defect (50). Therefore, while the absence of m^7^G alone may not be sufficient to force the tRNA structure away from its favored conformation, it is possible that this equilibrium could be disturbed under stress, exposing other modifications to an unfavorable environment or preventing their corresponding trms from making the modification. One potential example of an affected modification could be the Gm18 TrmH modification that was shown to reduce the immunostimulatory properties of bacterial tRNA in *E. coli* by suppressing TLR7-mediated interferon release (25, 50). Even if the other modifications on the tRNA are not affected by a disturbed equilibrium state in the absence of m^7^G stabilization, the very nature of a change in tRNA folding may be enough of a stressor to disrupt essential processes and reduce pathogenic potential.

Notably, the regulation of bacterial TrmB remains largely unknown, and its catalytic mechanism has still not been definitively proven, though several mechanisms have been proposed and catalytic residues have been identified (53). Furthermore, TrmB has been reported to dimerize in some bacteria and may require a partner protein in yeast, highlighting the mechanistic variation between species (38, 54). Potentially, bioinformatic analyses on the differentially regulated proteins could reveal that their transcripts are enriched for particular codons that would suggest putative tRNA species targeted by *A. baumannii* TrmB. This information would facilitate the identification of potential MoTTs that are regulated in response to the presence of certain stressors. Moreover, we aim to investigate whether *trmB* is being regulated pre or post-transcriptionally and if TrmB itself is regulating MoTTs post-transcriptionally. These analyses are imperative to increase our understanding of the role TrmB plays in *A. baumannii* pathogenicity.

In this paper, we discovered that TrmB is essential for *A. baumannii* oxidative and acid stress responses *in vitro*, replication within macrophages, and full virulence in a murine pneumonia model. As the *A. baumannii* vacuole is still being characterized, the physiologically relevant concentration of hydrogen peroxide in the vacuole is unknown. *In vitro* hydrogen peroxide killing assays with *A. baumannii* have been performed by multiple groups at concentrations ranging from 5 to 175 mM. In this work, we used 2–5 mM for our assays, which is at the lowest end of this range (29, 42, 55 – 57). To examine the role of TrmB *in* vivo, we utilized an additional clinical isolate, Ab04, to determine if TrmB is important for murine CAUTI and found that the presence of TrmB did not affect virulence in this model. This suggests that TrmB may play a bigger role in response to stressors imposed during pneumonia infections as compared to CAUTIs. In murine pneumonia models, macrophages play an integral role in eliminating *A. baumannii* infections, and modern clinical isolates can infect alveolar macrophages and replicate within vacuoles *in vivo* (33, 58). However, the role of macrophages in CAUTIs is still unknown, and no evidence has been found that *A. baumannii* is internalized in macrophages during CAUTIs. Therefore, TrmB may either be more important for *A. baumannii*-induced pneumonia or may regulate different virulence factors in clinical isolates that establish CAUTIs.

Beyond TrmB, we identified eight other putative SAM-dependent trms in the core genome of *A. baumannii*. Of these nine, two putative trms, TrmZ1 and TrmZ2, do not have an annotated modification; Phyre2 fold recognition analysis predicted TrmZ1 as a uracil-5 methyltransferase and TrmZ2 as either a 2′-O-methyltransferase or a guanine-N7 methyltransferase. However, as discussed previously, homology does not guarantee function. It is possible that there are more modifications and nonorthologous enzymes in *A. baumannii* that our analysis did not detect. In the future, we aim to use mass spectrometry to identify the full tRNA modificome of *A. baumannii* and elucidate its role in *A. baumannii* pathobiology.

*A. baumannii* is categorized at the highest threat level by the Centers for Disease Control and Prevention and the World Health Organization due to its increasing rates of MDR that far outpace other prominent Gram-negative pathogens (2, 3, 59). The ability of *A. baumannii* to successfully detect and respond to stress is integral to its success as a pathogen and to its survival in the environment, including on medical surfaces. While some recent studies have begun to explore the role of the tRNA modificome in bacteria, most research is still performed in model, domesticated strains, which have been shown to possess less or different stress response factors than modern clinical isolates (5, 60). *A. baumannii* in particular has a large accessory genome, with clinical strains only possessing part of a massive array of virulence factors (61). Therefore, the MoTTs that are impacted by a particular tRNA modification can vary between isolates. While the affected stress factors may vary, if all isolates are affected by the loss of trms in some aspect, then trms are still a viable drug target. Several groups are currently attempting to target TrmD therapeutically, as the modification that it catalyzes is essential to all domains of life (21 – 23). In fact, while many tRNA modifications are conserved across kingdoms, the catalytic mechanisms of the trms may not be, which is ideal for targeting bacterial trms without disturbing the function of the human trm equivalents. While TrmB is not essential in ideal conditions, we have shown that it is critical to *A. baumannii* virulence in a pneumonia model, which is one of the most common forms of *A. baumannii* infection in clinical settings (5). Ultimately, characterization of *A. baumannii* trms may prove to be a valuable tool for developing novel therapeutics to fight the meteoric rise of MDR *A. baumannii*.

## MATERIALS AND METHODS

### Bacterial strains and growth conditions

The strains and plasmids used in this study are described in Tables S1 and S2. Cultures were grown at 37°C and 200 rpm using lysogeny broth (LB) medium. When appropriate, *E. coli* strains were grown in the following antibiotic concentrations: 30 µg/mL apramycin or 50 µg/mL zeocin; and *A. baumannii* strains were grown in 50 µg/mL apramycin, 300 µg/mL hygromycin B, or 50 µg/mL zeocin. For phenotypic assays, strains were struck out on solid LB plates, grown overnight without antibiotics, subcultured and grown to mid-exponential phase (1 OD of mid-exponential culture ~4 × 10^8^ CFU/mL). Iron deprivation killing assays were performed with TMS medium or Chelex-100 treated TMS (cTMS) as previously described (62, 63).

### Construction of *trmB* mutants and complemented strains

The primers used in this study are listed in Table S3. The ARC6851 and Ab04 deletion mutants were constructed as previously described (64). DNA assembly was performed with the NEBuilder HiFi DNA Assembly Cloning Kit. Briefly, 1 kb regions upstream and downstream of genes to be deleted were amplified from ARC6851 genomic DNA (or Ab04 genomic DNA for Ab04 Δ*trmB*). Overlap extension PCR was used to fuse the fragments with an FRT site-flanked apramycin resistance cassette that was amplified from a variant of pKD4 (65, 66). The purified linear DNA product was electroporated into the proper wild-type strain containing the pAT04 plasmid that encodes an IPTG-inducible copy of RecAB recombinase, and mutants were selected using resistance to apramycin treatment (64). To excise the antibiotic cassette, a plasmid encoding an IPTG-inducible copy of FLP recombinase, PAT03, was transformed into each of the mutants (64). Clean mutants were confirmed via return of apramycin sensitivity and whole-genome sequencing. Genetic complementation of the mutants was performed with a mini-Tn7 system as previously described (67, 68). ARC6851 Δ*trmB* was complemented using pUCT18T-miniTn7-Zeo, and Ab04 Δ*trmB* was complemented using pUCT18T-miniTn7-Apra (69). Briefly, the plasmids were amplified and fused with the appropriate ARC6851 or Ab04 alleles along with ~500 bp upstream that contains the putative promoter region (65). The copy of the gene and the antibiotic resistance cassette were introduced into the chromosome of their respective mutant strains with four-parental conjugation and confirmed with PCR analyses (67 – 70). Complemented strains were confirmed using PCR and whole-genome sequencing.

### Bioinformatic analyses

BLAST was used to identify putative SAM-dependent trms using homology to known trms in other bacterial species, including *E. coli*, *P. aeruginosa, Pyrococcus abyssi*, and *Methanopyrus kandleri*. The locus tags of the identified trms are listed in Table 1.

### Antibiotic susceptibility assays

MICs were determined using the 2-fold broth dilution microtiter assay as previously described (71, 72). Overnight cultures were subcultured into 10 mL LB at an OD_600_ of 0.05 and grown for 3 h at 37°C and 200 rpm. About 10^5^ cells/mL of mid-exponential phase cells were used to inoculate a 96-well microtiter plate (Corning 3788) containing 2-fold dilutions of the appropriate antibiotics. Microtiter plates were incubated at 37°C under shaking conditions for 16 h before MICs were determined using OD_600_. MICs were defined as <10% growth compared to the non-treated controls. Experiments were performed at least five independent times.

### H_2_O_2_ killing assays

H_2_O_2_ killing assays were performed as previously described (42). Briefly, bacterial cultures were started from LB agar plates, inoculated in LB broth, and grown for 16 h at 37°C and 200 rpm. Overnight cultures were subcultured in 10 mL LB at OD_600_ 0.05 and incubated at 37°C and 200 rpm for 3 h to mid-exponential phase. Cultures were normalized to the lowest OD_600_, and 1 mL was aliquoted into 14 mL polypropylene round-bottom tubes (Falcon 352059). Cultures were treated with 0 or 5 mM H_2_O_2_ (Supelco HX0635-3) for 2 h at 37°C and 200 rpm. Killing was determined by samples being serially diluted and plated on LB agar, incubating the plates overnight, and enumerating CFU. In the iron deprivation experiments, mid-exponential cultures were normalized, washed two times in either LB broth, TMS, or cTMS, and treated with 0, 2, or 3 mM H_2_O_2_ as previously described (Fig S6). Experiments were performed at least five independent times.

### Growth curve assays

Growth curves were performed in sterile, round-bottom, polystyrene, 96-well plates (Corning 3788). Bacterial cultures were started from LB agar plates, inoculated in LB broth, and grown 16 h at 37°C and 200 rpm. Overnight cultures were subcultured in 10 mL LB at OD_600_ 0.05 and incubated at 37°C and 200 rpm for 3 h to mid-exponential phase. Cultures were diluted in fresh medium to an OD_600_ of 0.01 and inoculated into 96-well plates at a final volume of 150 µL. For acidic growth conditions, non-buffered LB was adjusted to pH 5.0 using HCl. Plates were incubated at 37°C in shaking conditions for 16 h in a BioTek microplate reader, with OD_600_ values measured at 30 min intervals. All experiments were performed on at least five independent days with at least three wells per strain per condition. Endpoint pH measurements were obtained as described previously (33).

### Cell culture conditions

The J774A.1 mouse macrophage cell line (ATCC TIB-67) was cultured in Dulbecco’s modified Eagle medium (DMEM) High Glycose (Hyclone, SH30022.01) supplemented with 10% heat-inactivated fetal bovine serum (FBS, Corning) at 37°C and 5% CO_2_.

### Intracellular replication assays

J774A.1 cells were seeded in 48-well plates 16 h before the experiment at 3 × 10^5^ cells/well and incubated at 37°C and 5% CO_2_. Bacterial cultures were started from LB agar plates, inoculated in LB broth, and grown for 16 h at 37°C and 200 rpm. Overnight cultures were subcultured in 10 mL LB at OD_600_ 0.05 and incubated at 37°C and 200 rpm for 3 h to mid-exponential phase. Cultures were washed two times with phosphate-buffered saline (PBS) and an appropriate volume was added to each well with DMEM + 10% FBS media to reach a multiplicity of infection (MOI) of 10. Plates were centrifuged 10 min at 200 × *g* to enhance bacterial contact with the host cells and incubated for 1 h at 37°C and 5% CO_2_. Wells were washed three times with PBS and fresh DMEM + 10% FBS supplemented with 30 µg/mL apramycin was added to the cells to eliminate extracellular bacteria. At 2, 4, and 6 h post-infection, the cells were washed three times with PBS and lysed with 500 µL PBS containing 0.05% Triton X-100. Lysates were serially diluted and plated on LB plates supplemented with chloramphenicol to determine CFU.

### Immunofluorescence staining

A total of 1.5 × 10^5^ J774A.1 cells were plated onto glass coverslips in 24-well plates and incubated 16 h at 37°C and 5% CO_2_. Bacterial inocula was prepared as described for intracellular replication assays. Once the bacterial suspensions were added to the wells at an MOI of 10, the plates were centrifuged at 200 × *g* and incubated at 37°C and 5% CO_2_ for 1 h. Then, the cells were washed three times with PBS and extracellular bacteria were killed with DMEM + 10% FBS supplemented with 50 µg/mL colistin. At 4 h, samples were washed with PBS and fixed with 4% paraformaldehyde for 15 min at 37°C and then incubated for 30 min shaking at room temperature in permeabilizing and blocking solution (PBS + 0.1% saponin + 0.5% BSA [Fisher BioReagents, BP9706100] and 10% FBS [Corning]). Then, the glass coverslips were incubated overnight at 4°C with primary antibodies developed against the insoluble fraction of *A. baumannii* and produced in rabbit. The next day, the coverslips were washed three times with washing solution (PBS + 0.1% saponin + 0.5% BSA) and incubated with DAPI, Alexa Fluor 555 phalloidin (0.33 µM; CST, #8943) at a 1:100 dilution, and a goat anti-rabbit secondary antibody Alexa Fluor 647 (Invitrogen, A-21244) at a 1:250 dilution for 1 h at 37°C. Afterward, coverslips were washed three times in PBS with washing solution, rinsed with water, and mounted on a glass slide in Invitrogen ProLong Gold Antifade Mountant (Invitrogen, P36930). Stained samples were analyzed by confocal microscopy.

### Confocal microscopy

Stained samples were analyzed with a Zeiss LSM880 laser scanning confocal microscope (Carl Zeiss Inc.) equipped with 405 nm diose, 488 nm, Argon, 543 nm HeNe, and 633 nm HeNe lasers. A Plan-Apochromat 63× (NA 1.4) DIC objective and ZEN black 2.1 SP3 software were used for image acquisition. Images were analyzed using ImageJ software (NIH, USA).

### Transmission electron microscopy

For quantitation of length, two biological replicates of each strain were subcultured and grown to mid-exponential growth as described previously. For negative staining and analysis by transmission electron microscopy, bacterial suspensions were allowed to absorb for 10 min onto freshly glow-discharged Formvar/carbon-coated copper grids. The grids were washed in distilled water and stained for 1 min with 1% aqueous uranyl acetate (Ted Pella Inc., Redding, CA, USA). Excess liquid was gently removed, and grids were air-dried. Samples were viewed on a JEOL 1200EX transmission electron microscope (JEOL USA, Peabody, MA, USA) equipped with an AMT 8-MP digital camera (Advanced Microscopy Techniques, Woburn, MA, USA). Forty bacteria for each strain and replicate were chosen randomly and images were taken at a magnification of 3,000×. The length of each bacterium was determined using ImageJ 1.38 g (National Institutes of Health, USA, customized for AMT images).

### Murine pneumonia model

All animal experiments were approved by the Washington University Animal Care and Use Committee, and we have complied with all relevant ethical regulations. The murine pneumonia infections were performed as previously described for *A. baumannii* (35). Briefly, overnight cultures of bacteria were subcultured into 100 mL of OD_600_ 0.05 and grown at 37°C and shaking for 3 h to mid-exponential growth. Cultures were washed two times and resuspended in PBS. Six- to 8-week-old female C57BL/6 mice (Charles River Laboratories, Wilmington, MA, USA) were anesthetized by inhalation of 4% isoflurane, and then intranasally inoculated with 5 × 10^7^ CFU of resuspended bacteria. At 24 h post-infection, the mice were sacrificed, and the lungs, kidneys, and spleens were aseptically removed. The bacterial load present in each tissue was determined by homogenizing each organ in PBS and plating serial dilutions on LB agar supplemented with chloramphenicol.

### Murine CAUTI model

The CAUTI infections were performed as previously described for *A. baumannii* (36). Briefly, bacteria were grown in static conditions at 37°C for 48 h, passaging once at 24 h. Six- to 8-week-old female C57BL/6 mice (Charles River Laboratories, Wilmington, MA, USA) were anesthetized by inhalation of 4% isoflurane, and a 4- to 5-mm piece of silicone tubing (catheter) was transplanted transurethrally. Cultures were washed two times and resuspended in PBS. Mice were infected immediately following implant placement with 50 µL containing ~1 × 10^8^ CFU of the bacterial suspension. At 24 h post-infection, the mice were sacrificed, and the catheters, bladders, and kidneys were harvested. The bacterial load present in each tissue was determined as previously described. Bacterial burden in catheters was determined by sonicating the catheters in PBS and plating as previously described.

### Measuring m^7^G levels in ARC6851

ARC6851 wild-type, Δ*trmB*, and *trmB*+ cultures were grown in LB for 16 h at 37°C and 200 rpm and then subcultured into 10 mL LB with an OD_600_ of 0.05. Three individual 10 mL culture biological replicates were prepared for each strain. Subcultures were grown for 3 h until midexponential. Samples were harvested by pelleting 2 mL OD_600_ 1.0, resuspending in Qiagen RNAprotect, incubating for 5 min, pelleting, removing the supernatant, and snap freezing. Samples were stored at −80°C until processing. Total RNA was extracted using the Zymo Research Quick-RNA Miniprep Kit. Sample quality was confirmed with Qubit RNA Broad Range and IQ Kits, and sample concentrations were normalized to 0.5 µg total RNA for digestion.

Free adenosine, guanosine, and cytosine standards were purchased from Acros Organics, uridine was purchased from Tokyo Chemical Industry, and m7G was purchased from Carbosynth. Samples were digested using nuclease P1 (Millipore Sigma, 10 units) at 50°C overnight (73). Afterwards, Tris pH 7.5 was added to a final concentration of 100 mM to adjust the pH, 10 units of CIP was added, and samples were incubated at 37°C for 90 min. After CIP treatment, samples were filtered using a 0.22-µM pore size syringe filter. To run each sample, 10 µL was loaded onto a Zorbax Eclipse Plus C18 column (2.1 × 50 mM, 1.8 µM) paired with an Agilent 6490 QQQ triple-quadrupole LC mass spectrometer. Runs were analyzed using multiple-reaction monitoring in positive-ion mode. The transitions used were: 268.1→136 (A), 244.1→112 (C), 284.2→152 (G), 245.1→113 (U), and 298→166 (m7G). Standard calibration curves were generated for each nucleoside by fitting the signal intensities against concentrations of pure-nucleoside preparations. The curves were used to determine the concentration of each respective nucleoside in the samples.

### Measuring m^7^G levels in Ab04

Total tRNA was purified from wild type, Δ*trmB*, and *trmB*+. Following purification, total tRNA was digested with ribonuclease T2 to generate 3′-monophosphate nucleotides and then vacuum-dried. The resulting pellet was resuspended in water and 5′-labeled with γ^32^P-ATP using T4 polynucleotide kinase to yield 3′,5′-diphosphate nucleotides, where the 5′-phospate was radioactive. Excess ATP was hydrolyzed using apyrase. The 3′-phosphate was cleaved using Nuclease P1 overnight (18 h) then vacuum-dried under high heat. The resulting pellet was resuspended in water then spotted on a cellulose TLC plate. The digested, 5′-labeled monophosphate nucleosides were separated in two dimensions. The first dimension was developed in solvent A {isobutyric acid/concentrated ammonia/water [66/1/33 (vol/vol/vol)]}. Once completely air-dried, the plate was rotated 90° and ran in solvent B {phosphate buffer/NH4 sulfate/n-propanol [100/60/2 (vol/wt/vol)]}. The dried plate was exposed to a PhosphorImager Screen overnight. Results were visualized using Typhoon FLA 9000 (GE) and analyzed using ImageQuant. Fraction of m7G was calculated using the signal from the pm7G spot divided by the total pm7G + pG signal (pm^7^G/pG + pm^7^G). Published two-dimensional TLC maps as well as all above methods are detailed further elsewhere (74, 75).

### Preparation of whole-cell pellets for comparative proteomics

ARC6851 wild-type, Δ*trmB*, and *trmB*+ cultures were grown in LB for 16 h at 37°C and 200 rpm. These overnight cultures were then subcultured into 10 mL LB at an OD_600_ of 0.05. Four individual 10 mL culture biological replicates were prepared for each condition: wild type (±H_2_O_2_), Δ*trmB* (±H_2_O_2_), and *trmB*+ (±H_2_O_2_). The subcultures were grown 3 h to mid-exponential and then either subjected to 2 mM H_2_O_2_ treatment or left untreated for 2 h growth at 37°C and 200 rpm. Whole cells were harvested by pelleting the 10 mL cultures at 4°C, washing with ice-cold PBS, and pelleting again before resuspending them in 4% SDS, 100 mM Tris pH 8.5.

### Sample preparation for proteomic analysis

Resuspended protein samples were solubilized by boiling them for 10 min at 95°C. The protein concentrations were then assessed by bicinchoninic acid protein assays (Thermo Fisher Scientific) and 200 µg of each biological replicate was prepared for digestion using Micro S-traps (Protifi, USA) according to the manufacturer’s instructions. Briefly, samples were reduced with 10 mM DTT for 10 min at 95°C and then alkylated with 40 mM IAA in the dark for 1 h. Samples were acidified to 1.2% phosphoric acid and diluted with seven volumes of S-trap wash buffer (90% methanol, 100 mM tetraethylammonium bromide, pH 7.1) before being loaded onto S-traps and washed three times with S-trap wash buffer. Samples were then digested with 2.5 µg of Trypsin overnight at 37°C before being collected by centrifugation with washes of 100 mM Tetraethylammonium bromide, followed by 0.2% formic acid, followed by 0.2% formic acid/50% acetonitrile. Samples were dried down and further cleaned up using C18 Stage tips to ensure the removal of any particulate matter (76, 77).

### Reverse phase liquid chromatography–mass spectrometry

C18 enriched proteome samples were resuspended in Buffer A × (2% acetonitrile, 0.01% trifluoroacetic acid) and separated using a two-column chromatography setup composed of a PepMap100 C_18_ 20 mm by 75 µM trap (Thermo Fisher Scientific) and a PepMap C_18_ 500 mm by 75 µm analytical column (Thermo Fisher Scientific) using a Dionex Ultimate 3000 UPLC (Thermo Fisher Scientific). Samples were concentrated onto the trap column at 5 µL/min for 6 min with Buffer A (0.1% formic acid, 2% DMSO) and then infused into an Orbitrap 480 (Thermo Fisher Scientific) at 300 nL/min via the analytical columns. Peptides were separated by altering the buffer composition from 3% Buffer B (0.1% formic acid, 77.9% acetonitrile, and 2% DMSO) to 23% B over 89 min, then from 23% B to 40% B over 10 min and then from 40% B to 80% B over 5 min. The composition was held at 80% B for 5 min before being returned to 3% B for 10 min. The Orbitrap 480 Mass Spectrometer was operated in a data-dependent mode automatically switching between the acquisition of a single Orbitrap MS scan (300–2,000 *m/z*, maximal injection time of 25 ms, an Automated Gain Control (AGC) set to a maximum of 300% and a resolution of 120k) and 3 s of Orbitrap MS/MS HCD scans of precursors (Stepped NCE of 25;30;45%, a maximal injection time of 80 ms, a AGC of 500% and a resolution of 30k).

### Proteomic data analysis

Identification and LFQ analysis were accomplished using MaxQuant (v1.6.17.0) using the ARC6851 (NCBI GCA_025677625.1/ASM2567762v1) with Carbamidomethyl (C) allowed as a fixed modification and Acetyl (Protein N-term) as well as Oxidation (M) allowed as variable modifications with the LFQ and “Match Between Run” options enabled (78). The resulting data files were processed using Perseus (version 1.6.0.7) with missing values imputed based on the total observed protein intensities with a range of 0.3 σ and a downshift of 1.8 σ (78). Statistical analysis was undertaken in Perseus using two-tailed unpaired *t* tests and ANOVAs. Matching of protein homologs between the strain ARC6851 and UPAB1 (NCBI GCF_006843645.1/ASM684364v1) and was undertaken using the proteome comparison tool within PATRIC, the bacterial bioinformatics database and analysis resource (79). Functional analysis was performed with eggNOG-mapper v2 (80).

### Reverse transcription-quantitative PCR

ARC6851 wild-type, Δ*trmB*, and *trmB*+ cultures were grown in LB for 16 h at 37°C and 200 rpm. These overnight cultures were then subcultured into 10 mL LB at an OD_600_ of 0.05 and grown for 3 h to mid-exponential phase. Cultures were treated with 0 mM or 2 mM H_2_O_2_ for 10 min or 2 h before being quickly pelleted, treated with RNAprotect (Qiagen, Inc.), and flash frozen. RNA was extracted from thawed samples using a TRIzol-chloroform extraction in conjunction with the Qiagen RNeasy Mini Kit. To remove contaminating DNA, both the Qiagen on-column DNase treatment and an off-column rigorous DNase treatment using the TURBO DNA-*free* kit were used. For reverse transcription (RT)-PCR, cDNA was prepared from 1 µg RNA using a high-capacity RNA-to-cDNA kit (Applied Biosystems), according to the manufacturer’s protocol. The cDNA was diluted to 20 ng/µL, and 1 µL was used as template for quantitative PCR (qPCR) using PowerUp SYBR green master mix (Applied Biosciences) on a ViiA7 real-time PCR machine (Applied Biosystems), following the manufacturer’s suggested protocol. The *A. baumannii rpoB* and *recA* genes were used as reference genes. All primers used for qPCR were designed using IDT PrimerQuest and are listed in Table S3. Threshold cycle (*C_T_*) values were normalized to the average of *rpoB* and *recA*, and fold changes and log_2_(fold changes) were calculated using the ΔΔ*C_T_* method.

### Statistical methods

All statistical analyses were performed using GraphPad Prism version 9.

## Data Availability

The mass spectrometry proteomics data have been deposited in the Proteome Xchange Consortium via the PRIDE partner repository with the data set identifier (81) PXD040002. This article contains supporting information (65, 69, 70, 82).

## AFTER EPUB

*[This article was published on 17 August 2023 with a typographical error in Results. The error was corrected in the current version, posted on 23 August 2023.]*

## SUPPLEMENTAL MATERIAL

Tables S1 and S2 mbio.01416-23-s0001.docx Plasmids and strains used in this study.

Supplemental Figures mbio.01416-23-s0002.docx Figures S1 to S6.

Data Set Legend mbio.01416-23-s0003.docx Legend for the proteomic data set.

Supplemental Data Set mbio.01416-23-s0004.xlsx Proteomic data set.

Table S3 mbio.01416-23-s0005.docx Primers used in this study.

Table S4 mbio.01416-23-s0006.docx Differentially expressed proteins in ARC6851 Δ*trmB* vs. wild type in LB.

Table S5 mbio.01416-23-s0007.docx Differentially expressed proteins in ARC6851 Δ*trmB* vs. wild type in H_2_O_2_.

ASM does not own the copyrights to Supplemental Material that may be linked to, or accessed through, an article. The authors have granted ASM a non-exclusive, world-wide license to publish the Supplemental Material files. Please contact the corresponding author directly for reuse.
