# Supplementary material for: The tRNA methyltransferase TrmB is critical for Acinetobacter baumannii stress responses and pulmonary infection
Source: mBio. 2023 Aug 17;14(5):e01416-23. doi: 10.1128/mbio.01416-23 (PMC10653896; doi:10.1128/mbio.01416-23)
Supplement: Tables S1 and S2 — Plasmids and strains used in this study. [file mbio.01416-23-s0001.docx]

| **Plasmid** | **Description^a^** | **Source** |
| --- | --- | --- |
| pKD4-Zeo | Source for zeocin cassette for mutant generation, Zeo^r^ | (1, 2) |
| pKD4-Apr | Source for Apramycin cassette for mutant generation, Apr^r^ | This study |
| pUCT18T-miniTn7T-Zeo | mTn7 complementation vector, Zeo^r^ | (3) |
| pUCT18T-miniTn7T-Apr | mTn7 complementation vector, Apr^r^ | This study |
| pUCT18T-miniTn7T-Zeo-ARC_trmB | ARC6851 Δ*trmB* complementation construct, Zeo^r^ | This study |
| pUCT18T-miniTn7T-Apr-Ab04_trmB | Ab04 Δ*trmB* complementation construct, Apr^r^ | This study |

**Table S1: Plasmids used in this study**

^a^Zeo, zeocin; Apr, apramycin

**Table S2: Strains used in this study**

| **Strain** | **Description** | **Source** |
| --- | --- | --- |
| *E. coli* Stellar | *mrr*-*hsdRMS*-*mcrBC* and *mcrA* | TaKaRa |
| *E. coli* HB101 | pRK2013 | (4) |
| *E. coli* EC100D | pTNS2 | (4) |
| ARC6851 | Wildtype | Entasis Therapeutics |
| ARC6851 Δ*trmA* | ARC6851 *trmA* mutant | This study |
| ARC6851 Δ*trmB* | ARC6851 *trmB* mutant | This study |
| ARC6851 *trmB+* | ARC6851 *trmB* mutant complemented | This study |
| ARC6851 Δ*mnmC* | ARC6851 *mnmC* mutant | This study |
| ARC6851 Δ*trmJ* | ARC6851 *trmJ* mutant | This study |
| ARC6851 Δ*trmL* | ARC6851 *trmL* mutant | This study |
| ARC6851 Δ*trmO* | ARC6851 *trmO* mutant | This study |
| ARC6851 Δ*trmZ1* | ARC6851 *trmZ1* mutant | This study |
| ARC6851 Δ*trmZ2* | ARC6851 *trmZ2* mutant | This study |
| Ab04 | Wildtype | (5) |
| Ab04 Δ*trmB* | Ab04 *trmB* mutant | This study |
| Ab04 *trmB+* | Ab04 *trmB* mutant complemented | This study |

**References**

1. Datsenko, K. A., and Wanner, B. L. (2000) One-step inactivation of chromosomal genes in Escherichia coli K-12 using PCR products. *Proc. Natl. Acad. Sci. U. S. A.* **97**, 6640–6645

2. Le, N. H., Peters, K., Espaillat, A., Sheldon, J. R., Gray, J., Venanzio, G. Di, Lopez, J., Djahanschiri, B., Mueller, E. A., Hennon, S. W., Levin, P. A., Ebersberger, I., Skaar, E. P., Cava, F., Vollmer, W., and Feldman, M. F. (2020) Peptidoglycan editing provides immunity to Acinetobacter baumannii during bacterial warfare. *Sci. Adv.* 10.1126/SCIADV.ABB5614

3. Ducas-Mowchun, K., De Silva, P. M., Crisostomo, L., Fernando, D. M., Chao, T. C., Pelka, P., Schweizer, H. P., and Kumar, A. (2019) Next generation of Tn7-based single-copy insertion elements for use in multi- and pan-drug-resistant strains of Acinetobacter baumannii. *Appl. Environ. Microbiol.* 10.1128/AEM.00066-19/SUPPL_FILE/AEM.00066-19-S0001.PDF

4. Kumar, A., Dalton, C., Cortez-Cordova, J., and Schweizer, H. P. (2010) Mini-Tn7 vectors as genetic tools for single copy gene cloning in Acinetobacter baumannii. *J. Microbiol. Methods*. **82**, 296–300

5. Ahmed-Bentley, J., Chandran, A. U., Joffe, A. M., French, D., Peirano, G., and Pitout, J. D. D. (2013) Gram-negative bacteria that produce carbapenemases causing death attributed to recent foreign hospitalization. *Antimicrob. Agents Chemother.* **57**, 3085–3091
