# Supplementary material for: The tRNA methyltransferase TrmB is critical for Acinetobacter baumannii stress responses and pulmonary infection
Source: mBio. 2023 Aug 17;14(5):e01416-23. doi: 10.1128/mbio.01416-23 (PMC10653896; doi:10.1128/mbio.01416-23)
Supplement: Supplemental Figures — Figures S1 to S6. [file mbio.01416-23-s0002.docx]

**
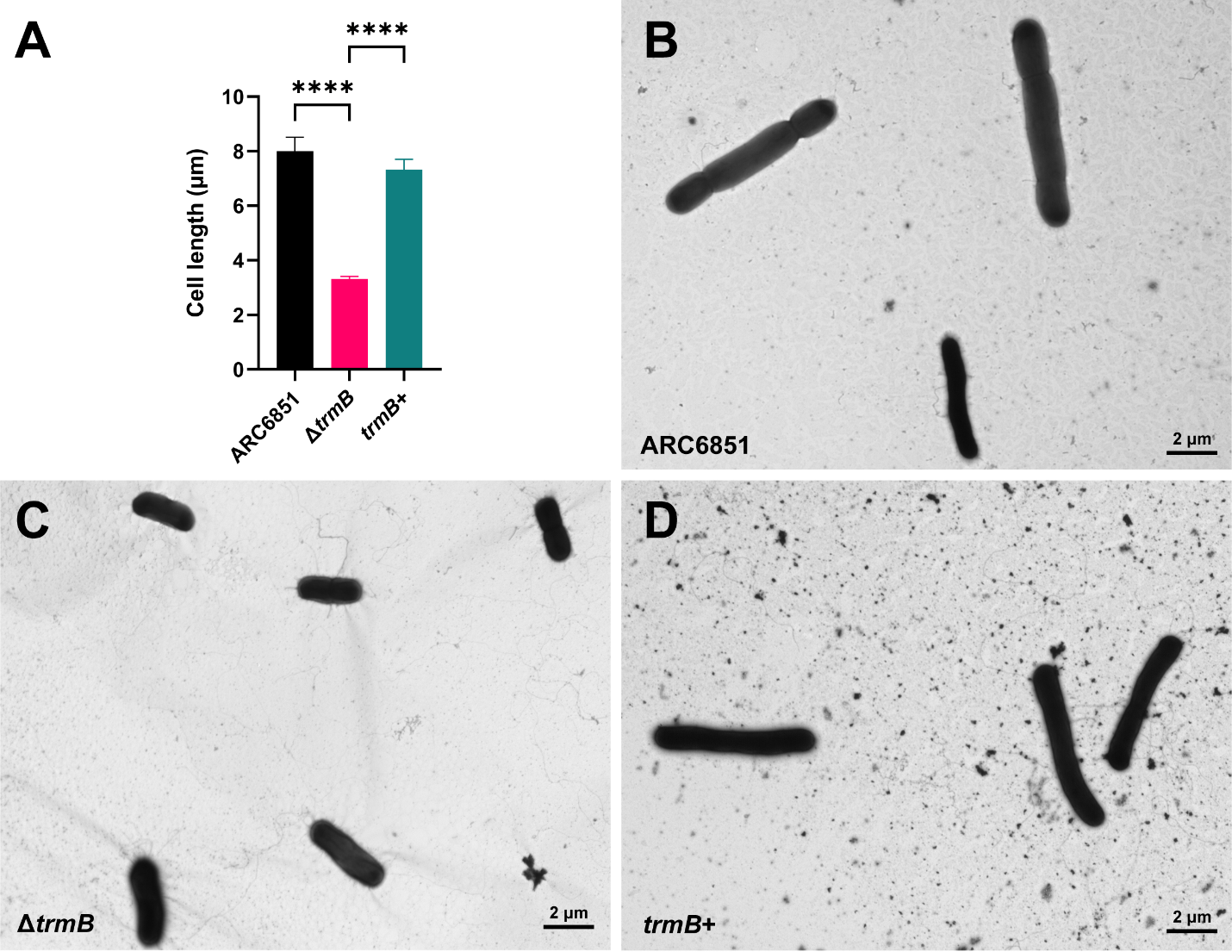
**

**Figure S1: ARC6851 Δ*trmB* is shorter in length than wildtype at mid-exponential growth** (A) Cell lengths quantified from transmission electron microscopy images of 80 cells and two biological replicates of ARC6851 wildtype, Δ*trmB*, and *trmB*+ strains grown at mid-exponential phase. Lengths were quantified with ImageJ 1.38g. *****P*<0.0001, one-way ANOVA, Tukey’s test for multiple comparisons. (B-D) Representative transmission electron microscopy images of ARC6851 wildtype (B), Δ*trmB* (C), and *trmB*+ (D) strains.

**
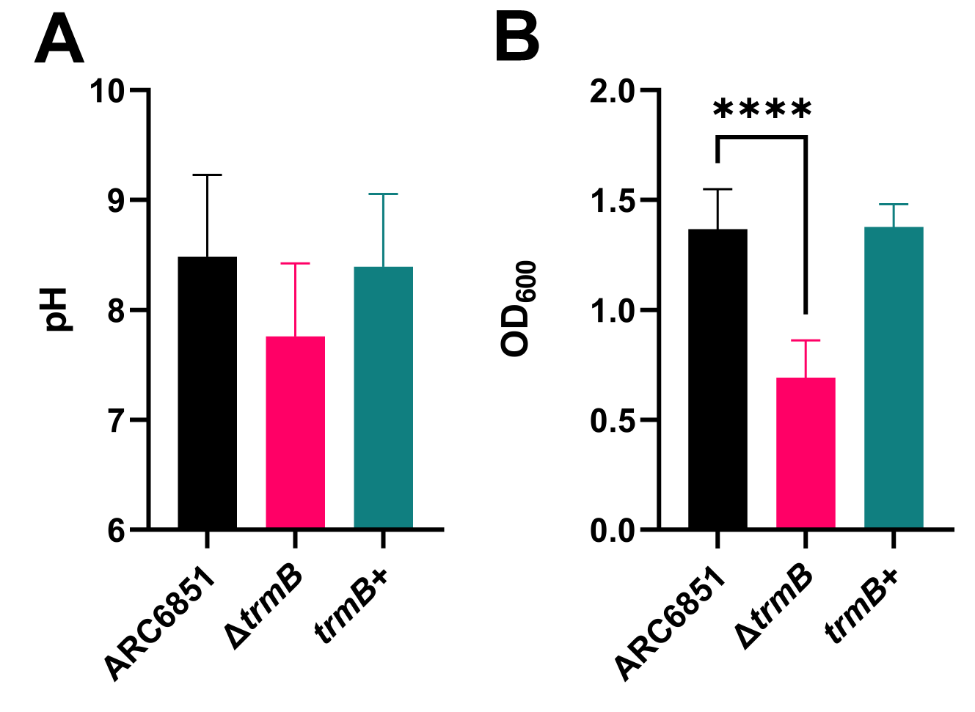
**

**Figure S2: ARC6851 Δ*trmB* may have a lower pH at late stationary phase in acidic media, likely due to its growth defect** (A) pH measured at 16 h of growth for ARC6851 wildtype, Δ*trmB*, and *trmB*+ strains. Bacterial cultures were grown in non-buffered LB pH 5.0 with phenol red (15 µg/mL), and OD_560_ and OD_600_ were measured at 16 h. (B) Bacterial density measured at 16 h of growth with OD­­­_600_. *****P*<0.0001, one-way ANOVA, Tukey’s test for multiple comparisons.

**
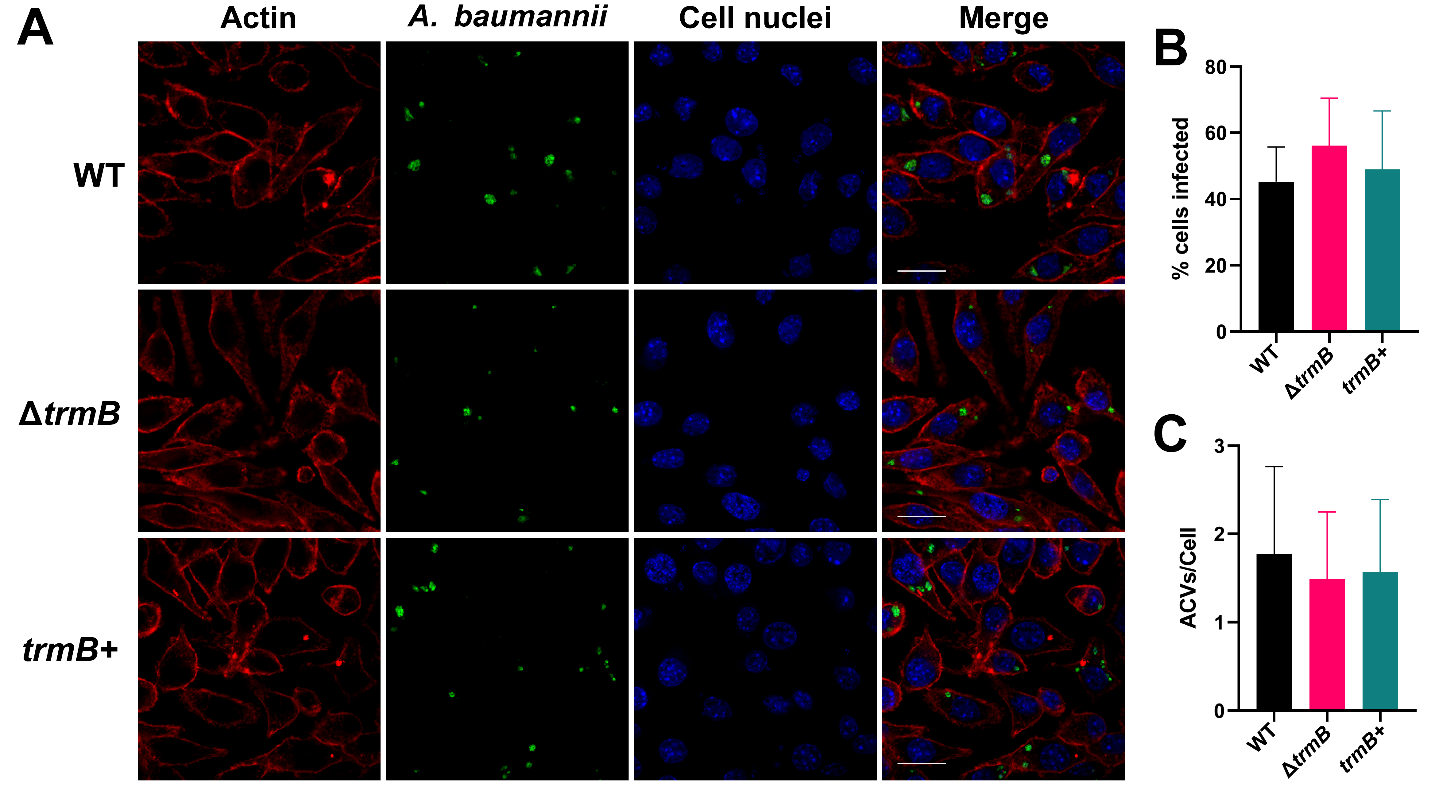
**

**Figure S3: The Δ*trmB* mutant has similar rates of infection and vacuoles formed per macrophage compared to wildtype** (A) J77A.1 macrophages infected with mid-exponential ARC6851 wildtype, Δ*trmB*, and *trmB*+ strains. Cells were fixed at 4 h post-infection, and the samples were stained to detect cell nuclei (blue), *A. baumannii* (green), and actin (red). Bars 20 μm. (B) At least 14 representative images per strain were analyzed. The number of cells infected did not differ between strains. (C) The number of ACVs per cell did not differ between strains.

**
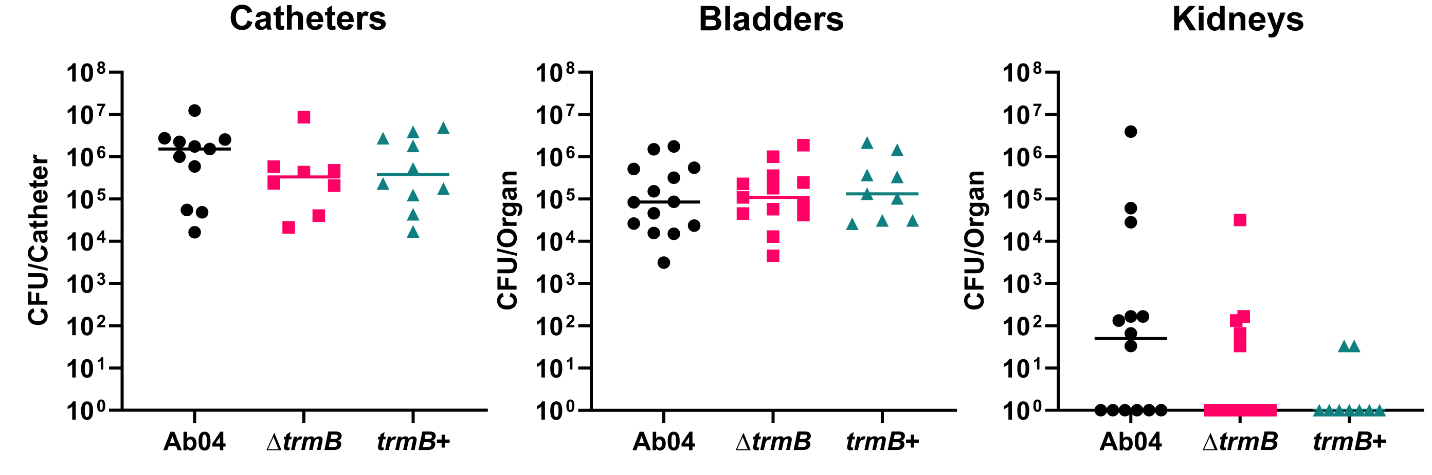
**

**Figure S4: Ab04 Δ*trmB* does not have an infection defect in a CAUTI murine model** Mice with an implanted catheter were transurethrally infected with ~1x10^8^ CFU of the indicated strains. At 24 h post-infection, bacterial burden of the catheter, bladder, and kidneys were determined. Each symbol represents an individual mouse. The horizontal line represents the median for each strain. Results shown from two pooled experiments.

**
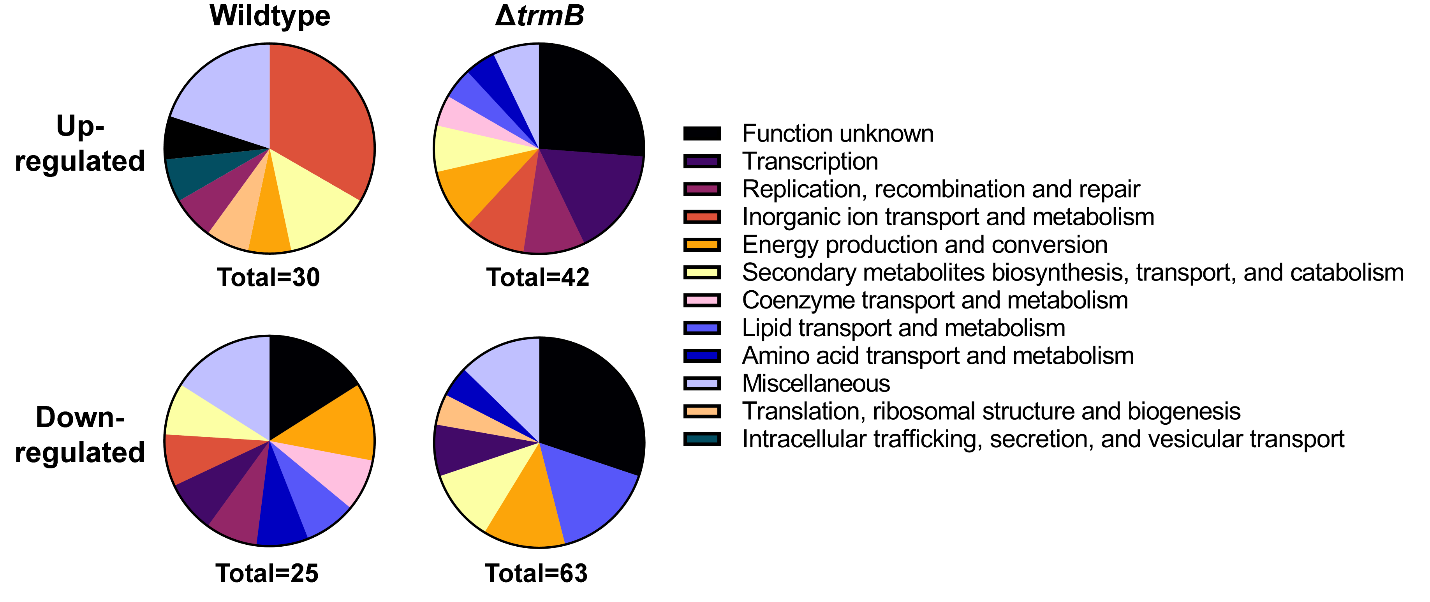
**

**Figure S5: TrmB mediates global changes in translation with and without oxidative stress** Functional analysis of whole cell proteomic data of ARC6851 wildtype and Δ*trmB* in H_2_O_2_ treatment compared to non-treated (**Table 2 and 3**). Functional annotation was completed on proteins with a log_2_fold change greater or equal to 1.00 with EggNOG-mapper v2 and charted with GraphPad Prism v9.

**
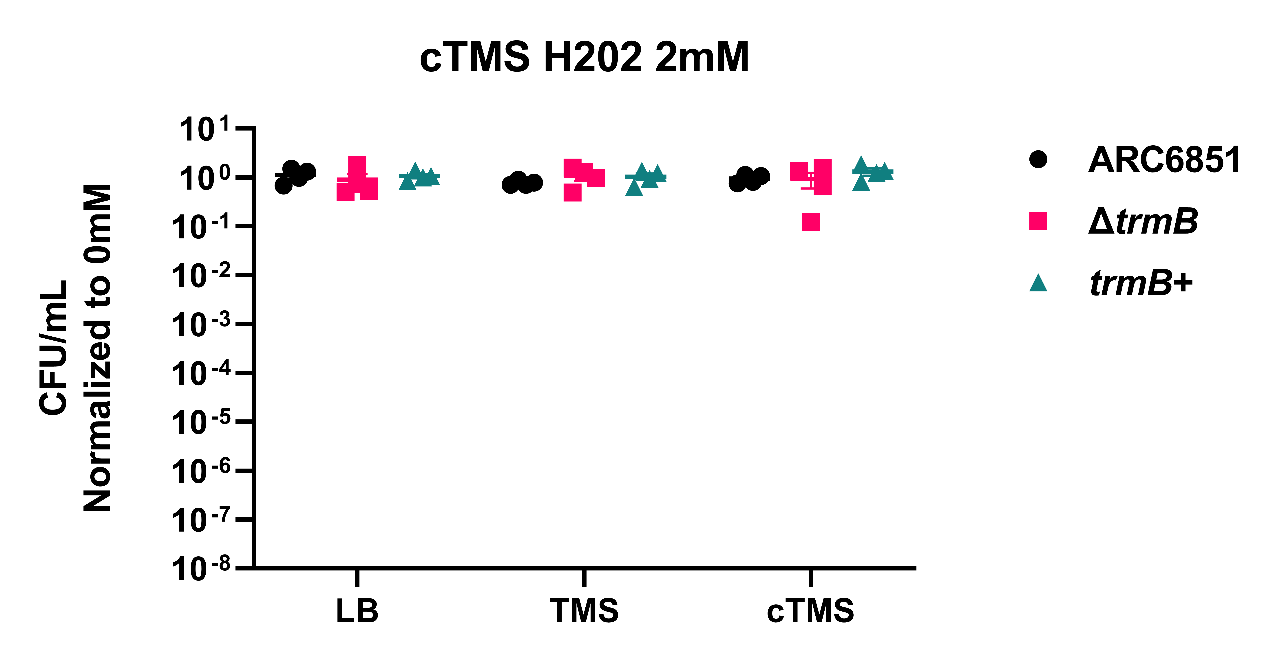
**

**Figure S6: ARC6851 Δ*trmB* is not more susceptible to 2mM H_2_O_2_ in chelated media** H_2_O_2_ killing of ARC6851 wildtype, Δ*trmB*, and *trmB*+ strains in grown in LB, Tris minimal succinate (TMS) media, or Chelex-100-treated TMS (cTMS) with treatment in 2 mM H_2_O_2_ for 2 h compared to 0 mM. Survival was measured by serial dilution and quantification of recoverable CFU/mL. Points represent technical replicates from at least 4 biological replicates, with a horizontal line representing the mean, and errors bars representing the standard error of the mean (SEM).
