# Supplementary material for: The tRNA methyltransferase TrmB is critical for Acinetobacter baumannii stress responses and pulmonary infection
Source: mBio. 2023 Aug 17;14(5):e01416-23. doi: 10.1128/mbio.01416-23 (PMC10653896; doi:10.1128/mbio.01416-23)
Supplement: Data Set Legend — Legend for the proteomic data set. [file mbio.01416-23-s0003.docx]

**Supplemental Proteomic Dataset Legend:** Raw dataset included in the 'Full datset' tab. 'Tables' tab includes the full protein set for Tables 2,3,S4, and S5
