# Supplementary material for: The tRNA methyltransferase TrmB is critical for Acinetobacter baumannii stress responses and pulmonary infection
Source: mBio. 2023 Aug 17;14(5):e01416-23. doi: 10.1128/mbio.01416-23 (PMC10653896; doi:10.1128/mbio.01416-23)
Supplement: Table S3 — Primers used in this study. [file mbio.01416-23-s0005.docx]

**Table S3: Primers used in this study**

| **Primer name** | **Sequence** | **Purpose** |
| --- | --- | --- |
| ARC *trmA*_up_F | TTTTACATCATGGCAACCGC | Amplify upstream region of ARC6851 *trmA* (OB946_10490) |
| ARC *trmA*_up_R | CGAAGCAGCTCCAGCCTACACAATCGCTACTTCTAATGTAGGCGGTG |  |
| ARC *trmA*_down_F | CGGAATAGGAACTAAGGAGGATATTCATATGTATTCGTTGATCCTCCACG | Amplify downstream region of ARC6851 *trmA* (OB946_10490) |
| ARC *trmA*_down_R | TTATGCTACGCAGTCCGG |  |
| ARC *trmB* PATO_F1 | ATTGCTCACATTTGCACGTG | Amplify upstream region of ARC6851 *trmB* (OB946_12575) |
| ARC *trmB* PATO_up_R | CGAAGCAGCTCCAGCCTACACAATCGCTAACGTCACAATTTCACGGTG |  |
| ARC *trmB* PATO_down_F | CGGAATAGGAACTAAGGAGGATATTCATATGGTAACTAAGTTTGAACGCCG | Amplify downstream region of ARC6851 *trmB* (OB946_12575) |
| ARC *trmB* PATO_R2 | CAAAGCTTATTAGCCCAAGC |  |
| ARC *mnmC* KO up_F | TATAGAGATACGATGACATGCC | Amplify upstream region of ARC6851 *mnmC* (OB946_02580) |
| ARC *mnmC* KO up_R | CGAAGCAGCTCCAGCCTACACAATCGCTGCCATTTAAAAATACATGCCG |  |
| ARC *mnmC* KO down_F | CGGAATAGGAACTAAGGAGGATATTCATATGGTGAAGCTTATCCTGTTCCC | Amplify downstream region of ARC6851 *mnmC* (OB946_02580) |
| ARC *mnmC* KO down_R | CTTAATTGGGAATGTCATGCG |  |
| ARC *trmJ* PATO_F1 | CTTAGAAGCACTTATCCGTG | Amplify upstream region of ARC6851 *trmJ* (OB946_10840) |
| ARC *trmJ* PATO_up_R | CGAAGCAGCTCCAGCCTACACAATCGCTTTGACCATGACAATACGCAC |  |
| ARC *trmJ* PATO_down_F | CGGAATAGGAACTAAGGAGGATATTCATATGACCTTTACGCTTGCGTCG | Amplify downstream region of ARC6851 *trmJ* (OB946_10840) |
| ARC *trmJ* PATO_R2 | CTAAACCGACATGCAAGGG |  |
| ARC *trmL* KO up_F | AACTTGTATGCAAGCCCC | Amplify upstream region of ARC6851 *trmL* (OB946_03595) |
| ARC *trmL* KO up_R | CGAAGCAGCTCCAGCCTACACAATCGCTCTAAATGCAATTGAGCGCC |  |
| ARC *trmL* KO down_F | CGGAATAGGAACTAAGGAGGATATTCATATGCTTATCTAATGCAACTGCCG | Amplify downstream region of ARC6851 *trmL* (OB946_03595) |
| ARC *trmL* KO down_R | ATCTTTGGTATTCGCTGGC |  |
| ARC *trmO* PATO_F1 | TTCAATGACATAGTCGCCC | Amplify upstream region of ARC6851 *trmO* (OB946_06360) |
| ARC *trmO* PATO_up_R | CGAAGCAGCTCCAGCCTACACAATCGCTTTGACCAAATTAGGCTGACG |  |
| ARC *trmO* PATO_down_F | CGGAATAGGAACTAAGGAGGATATTCATATGTGAAAGACACATCGCTCG | Amplify downstream region of ARC6851 *trmO* (OB946_06360) |
| ARC *trmO* PATO_R2 | AATCGGGATTGTAGGCGC |  |
| ARC *trmMc* PATO_F1 | GTGTTTTCGAATTGGTGGC | Amplify upstream region of ARC6851 *trmZ1* (OB946_02910) |
| ARC *trmMc* PATO_up_R | CGAAGCAGCTCCAGCCTACACAATCGCTAAACGCTCAAAAGTCTGCTG |  |
| ARC *trmMc* PATO_down_F | CGGAATAGGAACTAAGGAGGATATTCATATGAGTTTGGTATTCATGCGGG | Amplify downstream region of ARC6851 *trmZ1* (OB946_02910) |
| ARC *trmMc* PATO_R2 | TTTAAAGCCAGTAGCCGAG |  |
| ARC *trmZ2* KO up_F | ATTGCGTTTTAGCCAGAGG | Amplify upstream region of ARC6851 *trmZ2* (OB946_16835) |
| ARC *trmZ2* KO up_R | CGAAGCAGCTCCAGCCTACACAATCGCTCCAATTTCTACACAAATCGGAG |  |
| ARC *trmZ2* KO down_F | CGGAATAGGAACTAAGGAGGATATTCATATGATTATGACCAAGCCTGAAGG | Amplify downstream region of ARC6851 *trmZ2* (OB946_16835) |
| ARC *trmZ2* KO down_R | TTAATACATCTTGCTTGACACC |  |
| Ab04 *trmB* KO_up _F | CCATTAAAAGACAATGCAGCC | Amplify upstream region of Ab04 *trmB* (ACX61_12415) |
| Ab04 *trmB* KO_up _R | CGAAGCAGCTCCAGCCTACACAATCGCTCGACGCATAAACGTCACAAT |  |
| Ab04 *trmB* KO_down _F | CGGAATAGGAACTAAGGAGGATATTCATATGGTGACTAAGTTTGAACGCCG | Amplify downstream region of Ab04 *trmB* (ACX61_12415) |
| Ab04 *trmB* KO_down _R | ATTGAAAACTATGATGCTGGC |  |
| P1 | AGCGATTGTGTAGGCTGGAGCTG | Amplify antibiotic cassette for overlap PCR |
| P2 | CATATGAATATCCTCCTTAGTTCCTATTCCG |  |
| qPCR rpoB_F | ACG GTA CTG AGC GTG TAA TC | qPCR primers for *rpoB* reference gene  (OB946_17310) |
| qPCR rpoB_R | TTA CCA CTT GAG TGG GTC TTA C |  |
| qPCR recA_F | GCC GTA TCT ACA GGT TCT TT | qPCR primers for *recA* reference gene  (OB946_08020) |
| qPCR recA_R | CCA GAA GAT TCA GGA CCA TAA |  |
| qPCR basB_F | GGC GGC CAT TCA CTA TTA | qPCR primers for *basB*  (OB946_05155) |
| qPCR basB_R | GGC AGC AGA TGG AGA TTT A |  |
| qPCR basE_F | GGC GTG GTT GTA CTC AAT | qPCR primers for *basE*  (OB946_05195) |
| qPCR basE_R | GCT GAC GCG AAC CAA TTA |  |
| qPCR bauA_F | TAT GCA TTA GGC ACC GTA TT | qPCR primers for *bauA*  (OB946_05180) |
| qPCR bauA_R | TGT TTG AGC AGT TTG TTG TT |  |
| qPCR bauB_F | GGG AGT AGC CCT CAT CAT A | qPCR primers for *bauB*  (OB946_05175) |
| qPCR bauB_R | AAT CGG TTC TGC TAG CTT C |  |
| qPCR OHRP_F | CAA CTT CAT CAG ACG GTG TA | qPCR primers for *OHRP* (OB946_15780) |
| qPCR OHRP_R | CTG CAA ATA ATT GTT CTG GGT |  |
