# Supplementary material for: The tRNA methyltransferase TrmB is critical for Acinetobacter baumannii stress responses and pulmonary infection
Source: mBio. 2023 Aug 17;14(5):e01416-23. doi: 10.1128/mbio.01416-23 (PMC10653896; doi:10.1128/mbio.01416-23)
Supplement: Table S4 — Differentially expressed proteins in ARC6851 ΔtrmB vs. wild type in LB. [file mbio.01416-23-s0006.docx]

**Table S4: Differentially expressed proteins in ARC6851 Δ*trmB* vs. wildtype in LB**

| **Accession** | **Fold change** | **Annotated protein** |
| --- | --- | --- |
| UYC75741.1 | **10.67** | SfnB family sulfur acquisition oxidoreductase |
| UYC76950.1 | 5.12 | preprotein translocase subunit SecE |
| UYC76690.1 | **3.05** | 2-amino-4-hydroxy-6-hydroxymethyldihydropteridine diphosphokinase |
| UYC76193.1 | 3.02 | hypothetical protein OB946_13215 |
| UYC76981.1 | **2.88** | ferrous iron transport protein A |
| UYC77666.1 | 2.86 | succinate dehydrogenase assembly factor 2 |
| UYC78960.1 | **2.72** | hypothetical protein OB946_09245 |
| UYC78228.1 | 2.65 | xanthine dehydrogenase molybdopterin binding subunit |
| UYC76774.1 | **0.40** | sel1 repeat family protein |
| UYC78103.1 | **0.40** | tRNA dihydrouridine synthase DusB |
| UYC77484.1 | **0.39** | hypothetical protein OB946_01155 |
| UYC76575.1 | 0.38 | cold shock domain-containing protein |
| UYC78848.1 | 0.38 | muconolactone Delta-isomerase |
| UYC76799.1 | 0.38 | RNA-binding protein |
| UYC78132.1 | **0.38** | type I-F CRISPR-associated helicase Cas3f |
| UYC77223.1 | **0.37** | sulfonate ABC transporter substrate-binding protein |
| UYC78830.1 | **0.37** | OprD family porin |
| UYC79094.1 | **0.36** | NAD-dependent deacylase |
| UYC75546.1 | **0.36** | dihydrolipoyl dehydrogenase |
| UYC76138.1 | **0.36** | AraC family transcriptional regulator |
| UYC79000.1 | **0.34** | anthranilate 1,2-dioxygenase electron transfer component AntC |
| UYC78998.1 | **0.33** | anthranilate 1,2-dioxygenase large subunit |
| UYC75996.1 | 0.33 | ferredoxin family protein |
| UYC75548.1 | **0.31** | alpha-ketoacid dehydrogenase subunit beta |
| UYC78999.1 | **0.30** | anthranilate 1,2-dioxygenase small subunit |
| UYC75549.1 | **0.30** | thiamine pyrophosphate-dependent dehydrogenase E1 component subunit alpha |
| UYC78834.1 | **0.25** | nuclear transport factor 2 family protein |
| UYC76640.1 | **0.25** | CoA pyrophosphatase |
| UYC78708.1 | **0.24** | hypothetical protein OB946_07885 |
| UYC77904.1 | **0.23** | nuclear transport factor 2 family protein |
| UYC77806.1 | **0.23** | VOC family protein |
| UYC75547.1 | **0.21** | 2-oxo acid dehydrogenase subunit E2 |
| UYC76625.1 | **0.19** | adenosine deaminase |
| UYC78695.1 | **0.19** | GTP 3,8-cyclase MoaA |
| UYC77015.1 | **0.09** | sulfonamide-resistant dihydropteroate synthase Sul1 |
| UYC78900.1 | **0.09** | TetR/AcrR family transcriptional regulator |
| UYC76077.1 | **0.08** | tRNA (guanosine(46)-N7)-methyltransferase TrmB |
| UYC78519.1 | **0.05** | aspartate/glutamate racemase family protein |

*Fold change cutoff: 2.5-fold, bolded if p-value < 0.05, Student’s unpaired *t*-test.
