# Supplementary material for: The tRNA methyltransferase TrmB is critical for Acinetobacter baumannii stress responses and pulmonary infection
Source: mBio. 2023 Aug 17;14(5):e01416-23. doi: 10.1128/mbio.01416-23 (PMC10653896; doi:10.1128/mbio.01416-23)
Supplement: Table S5 — Differentially expressed proteins in ARC6851 ΔtrmB vs. wild type in H2O2. [file mbio.01416-23-s0007.docx]

**Table S5: Differentially expressed proteins in ARC6851 Δ*trmB* vs. wildtype in H_2_O_2_**

| **Accession** | **Fold change** | **Annotated protein** |
| --- | --- | --- |
| UYC76950.1 | **9.83** | preprotein translocase subunit SecE |
| UYC78524.1 | **8.47** | TetR/AcrR family transcriptional regulator |
| UYC75741.1 | 7.37 | SfnB family sulfur acquisition oxidoreductase |
| UYC78650.1 | 5.34 | ATP-binding cassette domain-containing protein |
| UYC78239.1 | **4.35** | hypothetical protein OB946_05355 |
| UYC76578.1 | 4.26 | NADH-quinone oxidoreductase subunit M |
| UYC76045.1 | **4.22** | cold-shock protein |
| UYC77797.1 | **4.14** | hypothetical protein OB946_02925 |
| UYC76833.1 | **3.90** | MBL fold metallo-hydrolase |
| UYC76938.1 | **3.85** | TRAP transporter large permease subunit |
| UYC78238.1 | 3.28 | hypothetical protein OB946_05350 |
| UYC76426.1 | **3.28** | YfdQ family protein |
| UYC76360.1 | **3.26** | XRE family transcriptional regulator |
| UYC78273.1 | **3.16** | major capsid protein |
| UYC78262.1 | 3.00 | hypothetical protein OB946_05475 |
| UYC77357.1 | **2.93** | D-aminoacyl-tRNA deacylase |
| UYC77976.1 | 2.80 | diacylglycerol kinase |
| UYC78240.1 | 2.78 | ATP-binding protein |
| UYC79068.1 | 2.74 | PLP-dependent aminotransferase family protein |
| UYC78127.1 | 2.64 | DUF3144 domain-containing protein |
| UYC78349.1 | **2.64** | hypothetical protein OB946_05940 |
| UYC77609.1 | 2.59 | PspC domain-containing protein |
| UYC76769.1 | **2.57** | TonB-dependent siderophore receptor |
| UYC76715.1 | **2.55** | tRNA threonylcarbamoyladenosine dehydratase |
| UYC78055.1 | **2.54** | nicotinate-nicotinamide nucleotide adenylyltransferase |
| UYC77207.1 | **2.53** | 1,6-anhydro-N-acetylmuramyl-L-alanine amidase AmpD |
| UYC77517.1 | **0.40** | hypothetical protein OB946_01340 |
| UYC76622.1 | **0.40** | acyl-CoA dehydrogenase |
| UYC75836.1 | **0.40** | catalase HPII |
| UYC76570.1 | **0.40** | mechanosensitive ion channel |
| UYC76941.1 | 0.40 | YMGG-like glycine zipper-containing protein |
| UYC78475.1 | **0.40** | CoA-acylating methylmalonate-semialdehyde dehydrogenase |
| UYC76001.1 | **0.40** | IMPACT family protein |
| UYC76439.1 | **0.39** | FRG domain-containing protein |
| UYC78747.1 | **0.38** | universal stress protein |
| UYC76148.1 | **0.38** | nuclear transport factor 2 family protein |
| UYC75926.1 | **0.38** | phenylacetate-CoA oxygenase subunit PaaJ |
| UYC78302.1 | **0.38** | alpha/beta hydrolase |
| UYC76970.1 | **0.38** | TetR/AcrR family transcriptional regulator |
| UYC79148.1 | 0.38 | lysozyme inhibitor LprI family protein |
| UYC75544.1 | **0.37** | 2,3-butanediol dehydrogenase |
| UYC77996.1 | **0.37** | FdhF/YdeP family oxidoreductase |
| UYC78788.1 | **0.37** | hypothetical protein OB946_08315 |
| UYC76560.1 | **0.36** | hypothetical protein OB946_15200 |
| UYC78152.1 | **0.36** | thiamine pyrophosphate-binding protein |
| UYC78130.1 | **0.36** | DUF962 domain-containing protein |
| UYC78839.1 | **0.36** | flavin reductase family protein |
| UYC77859.1 | **0.35** | DUF1176 domain-containing protein |
| UYC78207.1 | **0.35** | siderophore-binding periplasmic lipoprotein BauB |
| UYC77949.1 | **0.35** | metalloprotease |
| UYC78999.1 | **0.35** | anthranilate 1,2-dioxygenase small subunit |
| UYC78834.1 | **0.35** | nuclear transport factor 2 family protein |
| UYC77223.1 | **0.35** | sulfonate ABC transporter substrate-binding protein |
| UYC78998.1 | **0.34** | anthranilate 1,2-dioxygenase large subunit |
| UYC77830.1 | **0.34** | YegP family protein |
| UYC79094.1 | **0.33** | NAD-dependent deacylase |
| UYC79075.1 | **0.33** | acyl-CoA dehydrogenase family protein |
| UYC78379.1 | **0.32** | hypothetical protein OB946_06090 |
| UYC78711.1 | **0.32** | D-amino acid dehydrogenase |
| UYC78474.1 | **0.32** | aspartate aminotransferase family protein |
| UYC77309.1 | **0.32** | DNA-3-methyladenine glycosylase I |
| UYC79244.1 | **0.32** | helix-turn-helix transcriptional regulator (plasmid) |
| UYC76080.1 | **0.32** | OmpA family protein |
| UYC76640.1 | **0.32** | CoA pyrophosphatase |
| UYC79000.1 | **0.31** | anthranilate 1,2-dioxygenase electron transfer component AntC |
| UYC77155.1 | **0.31** | CoA-acylating methylmalonate-semialdehyde dehydrogenase |
| UYC78211.1 | **0.31** | (2,3-dihydroxybenzoyl)adenylate synthase BasE |
| UYC78816.1 | **0.31** | protocatechuate 3,4-dioxygenase subunit alpha |
| UYC76571.1 | **0.31** | hypothetical protein OB946_15255 |
| UYC76653.1 | **0.31** | ribosome-associated translation inhibitor RaiA |
| UYC75917.1 | **0.30** | PaaI family thioesterase |
| UYC77692.1 | **0.29** | ribonuclease I |
| UYC77165.1 | 0.29 | ester cyclase |
| UYC77153.1 | **0.29** | AMP-binding protein |
| UYC77169.1 | **0.28** | SDR family NAD(P)-dependent oxidoreductase |
| UYC76799.1 | 0.27 | RNA-binding protein |
| UYC77084.1 | 0.27 | RidA family protein |
| UYC76604.1 | **0.27** | TetR/AcrR family transcriptional regulator |
| UYC77154.1 | **0.27** | 3-hydroxyisobutyrate dehydrogenase |
| UYC76238.1 | **0.25** | SRPBCC domain-containing protein |
| UYC78477.1 | 0.24 | DUF2171 domain-containing protein |
| UYC75753.1 | **0.24** | tautomerase family protein |
| UYC78031.1 | **0.24** | AI-2E family transporter |
| UYC76313.1 | **0.23** | biliverdin-producing heme oxygenase |
| UYC77971.1 | **0.22** | diacylglycerol kinase family protein |
| UYC78767.1 | **0.22** | hypothetical protein OB946_08205 |
| UYC78838.1 | **0.20** | PDR/VanB family oxidoreductase |
| UYC78208.1 | **0.20** | TonB-dependent siderophore receptor BauA |
| UYC77806.1 | **0.19** | VOC family protein |
| UYC78778.1 | **0.18** | Rieske (2Fe-2S) protein |
| UYC78876.1 | **0.18** | hydrolase |
| UYC75547.1 | **0.16** | 2-oxo acid dehydrogenase subunit E2 |
| UYC77015.1 | **0.15** | sulfonamide-resistant dihydropteroate synthase Sul1 |
| UYC78848.1 | **0.15** | muconolactone Delta-isomerase |
| UYC79121.1 | **0.14** | TonB-dependent receptor |
| UYC76876.1 | **0.13** | chaperone modulator CbpM |
| UYC75549.1 | **0.11** | thiamine pyrophosphate-dependent dehydrogenase E1 component subunit alpha |
| UYC75548.1 | **0.10** | alpha-ketoacid dehydrogenase subunit beta |
| UYC76077.1 | **0.07** | tRNA (guanosine(46)-N7)-methyltransferase TrmB |
| UYC78519.1 | **0.05** | aspartate/glutamate racemase family protein |
| UYC79050.1 | **0.02** | acinetobactin non-ribosomal peptide synthetase subunit BasB |

*Fold change cutoff: 2.5-fold, bolded if p-value < 0.05, Student’s unpaired *t*-test.
